# Population Genetics of *Trypanosoma brucei rhodesiense*: Clonality and Diversity within and between Foci

**DOI:** 10.1371/journal.pntd.0002526

**Published:** 2013-11-14

**Authors:** Craig W. Duffy, Lorna MacLean, Lindsay Sweeney, Anneli Cooper, C. Michael R. Turner, Andy Tait, Jeremy Sternberg, Liam J. Morrison, Annette MacLeod

**Affiliations:** 1 Wellcome Trust Centre for Molecular Parasitology, Institute of Biodiversity, Animal Health and Comparative Medicine, College of Medical, Veterinary and Life Sciences, University of Glasgow, Glasgow, United Kingdom; 2 Institute of Biological and Environmental Sciences, Zoology Building, University of Aberdeen, Aberdeen, United Kingdom; 3 Institute of Infection, Immunity and Inflammation, College of Medical, Veterinary and Life Sciences, University of Glasgow, Glasgow, United Kingdom; 4 Roslin Institute, University of Edinburgh, Easter Bush, Midlothian, United Kingdom; International Centre of Insect Physiology and Ecology, Kenya

## Abstract

African trypanosomes are unusual among pathogenic protozoa in that they can undergo their complete morphological life cycle in the tsetse fly vector with mating as a non-obligatory part of this development. *Trypanosoma brucei rhodesiense*, which infects humans and livestock in East and Southern Africa, has classically been described as a host-range variant of the non-human infective *Trypanosoma brucei* that occurs as stable clonal lineages. We have examined *T. b. rhodesiense* populations from East (Uganda) and Southern (Malawi) Africa using a panel of microsatellite markers, incorporating both spatial and temporal analyses. Our data demonstrate that Ugandan *T. b. rhodesiense* existed as clonal populations, with a small number of highly related genotypes and substantial linkage disequilibrium between pairs of loci. However, these populations were not stable as the dominant genotypes changed and the genetic diversity also reduced over time. Thus these populations do not conform to one of the criteria for strict clonality, namely stability of predominant genotypes over time, and our results show that, in a period in the mid 1990s, the previously predominant genotypes were not detected but were replaced by a novel clonal population with limited genetic relationship to the original population present between 1970 and 1990. In contrast, the Malawi *T. b. rhodesiense* population demonstrated significantly greater diversity and evidence for frequent genetic exchange. Therefore, the population genetics of *T. b. rhodesiense* is more complex than previously described. This has important implications for the spread of the single copy *T. b. rhodesiense* gene that allows human infectivity, and therefore the epidemiology of the human disease, as well as suggesting that these parasites represent an important organism to study the influence of optional recombination upon population genetic dynamics.

## Author Summary

Trypanosomes are single-celled organisms transmitted by the biting tsetse fly, which cause sleeping sickness in humans in sub-Saharan Africa, but also infect livestock and other mammals. Most trypanosomes cannot infect humans as they die in human serum, but two mutants of *Trypanosoma brucei* have evolved the ability to survive in human serum. This survival in human serum is conferred by the presence of one gene in the East African human-infective *T. b. rhodesiense*. How often trypanosomes exchange genetic material (they can mate in the tsetse fly) is debated, but will impact upon the spread of genes (e.g. that which confers human infectivity) through a population. We studied *T. b. rhodesiense* populations from different geographic locations (Malawi and two locations in Uganda), and over time (Uganda), to see if the populations are stable over time and space, using a panel of variable genetic markers enabling assessment of diversity. Our results suggest that there is significant difference in diversity between locations; those in Uganda are very closely related, increasingly so over time, whereas the Malawi population is very genetically diverse, consistent with the trypanosomes mating. These findings suggest that a greater understanding of *T. b. rhodesiense* population evolution will inform on sleeping sickness epidemiology.

## Introduction

Pathogens that can adapt quickly to environmental change often pose the greatest challenge to disease control. A clear example of this is the generation of drug resistance and subsequent rapid spread through a population [1]. The means and dynamics by which any trait spreads will depend upon the population structure and the level of recombination of the organism within individual populations. Therefore, understanding the population genetic dynamics of a pathogen and how often they share and disseminate genetic material is an important component in the development of risk assessment and intervention strategies.

The evolutionary potential of pathogen populations is a product of a number of factors, including the system of reproduction, the potential for gene flow, the effective population size and the mutation rate. Protozoan parasites offer a particular analytic challenge in this regard as many have complex life cycles in both vector and host, with some life cycle stages that expand mitotically and others in which sexual recombination occurs, resulting in mixed reproductive systems. Analyses of pathogenic protozoan populations reveal that there is significant diversity between different species and populations of the same species in terms of the role of genetic exchange, with some species showing clear clonality [2]–[4], while others demonstrate epidemic or panmictic populations. It is likely that the degree of recombination is dependent on local epidemiological factors [5]–[7]. Comprehensive analyses of multiple populations have been carried out for the malaria parasite, *Plasmodium falciparum*, which undergoes both asexual reproduction and an obligate sexual component of the life cycle, including out-crossing and self-fertilization. As sexual reproduction occurs in the insect vector, the frequency of out-crossing is a consequence of the transmission intensity, thus differences in transmission can result in a spectrum of population structures ranging from effective clonality (due to extensive self fertilization) to panmixia [8]. Thus there is a complex interaction between the epidemiology of the vector, host and parasite that influences the reproductive potential of the parasite. The *Plasmodium* research demonstrates that sampling from a range of epidemiological situations is necessary to evaluate the role of recombination in shaping the population genetic structure of a particular parasite species.

While mating in Apicomplexan parasites is an obligatory part of their life cycle in the arthropod vector, this is not the case with African trypanosomes. This issue is probably central to the controversy that has surrounded the definition of population structure and the role of mating in natural populations of the zoonotic protozoan parasite, *Trypanosoma brucei* [3], [9]–[11]. *T. brucei* is transmitted by tsetse flies (*Glossina* spp.) and in humans two subspecies, *T. b. rhodesiense* and *T. b. gambiense*, cause the often-fatal disease Human African Trypanosomiasis (HAT), also known as Sleeping Sickness. Sexual recombination in *T. brucei* occurs in the tsetse fly salivary glands and is well characterised under laboratory conditions [12]–[16]. Laboratory analysis has provided robust evidence that alleles segregate in a Mendelian manner [17] and the available data support the occurrence of both cross- and self-fertilisation [18], [19]. However, mating is not obligatory and does not happen with every transmission through a tsetse fly [20]. Thus, the parasite has the capacity for both clonal propagation with no sexual recombination, and also sexual propagation with varying degrees of inbreeding or out-crossing. This means that ‘clonality’ with respect to trypanosomes can be considered in two ways – that of classical mitotic clonality in the absence of sexual recombination [21], and the ‘reproductive clonality’ as has been observed in malaria parasites that undergo obligatory sexual recombination but in areas of both high and low transmission can undergo extensive inbreeding [22]–[24].

Initial isoenzyme analysis of *T. brucei* isolates from tsetse flies in East Africa indicated a panmictic or randomly mating population structure [9]. This interpretation was subsequently contested when high levels of linkage disequilibrium, lack of agreement with Hardy-Weinberg and the occurrence of identical genotypes at high frequency suggested either a clonal population structure where genetic exchange was very infrequent [2], [3], [25], or an epidemic population structure where there is a background level of frequent sexual recombination with the occasional clonal expansion of a few particular genotypes [26]. However, the interpretation of clonality is difficult with respect to trypanosomes, and counterarguments have centred on the existence of population sub-structuring, due either to geography or host specificity [27]. Genotype bias provided by the amplification of parasites *in vitro* or *in vivo* prior to analysis has also been suggested as another possible reason for the departures from expected genotype or allele frequencies [27], [28] and indeed this has been shown to occur [29]–[31].

An additional confounding factor for the study of *T. brucei* population genetics is that *T. brucei* consists of three morphologically identical sub-species. *T. b. brucei* cannot infect humans but causes disease in a wide range of domestic and wild animals, whereas *T. b. gambiense* is responsible for HAT in West and Central Africa, a chronic disease, and *T. b. rhodesiense* causes HAT in East and Southern Africa, typically a more acute disease. *T. b. gambiense* has been subdivided into two groups consisting of a homogeneous group 1 and a less common more heterogeneous group 2 [32]. Domestic and wild animals have been implicated as reservoirs of both human infective sub-species [33]–[35]. Several early studies failed to distinguish between the three sub-species and treated them as a single population, which may explain the detected high level of linkage disequilibrium [2], [3], [25]. From all available data it seems clear that *T. b. gambiense* group 1 is a clonal organism that undergoes sexual recombination very rarely, if at all [36], [37]. Indeed, *T. b. gambiense* group 1 is clearly genetically distinct from both *T. b. brucei* and *T. b. rhodesiense* [38]–[40]. Microsatellite analysis of 27 *T. b. rhodesiense* isolates from a range of foci in East and Southern Africa has shown that while isolates from different foci are broadly similar to each other, there is an association of the genotypes with their geographical origin [39]. However, the detailed analysis of the genetic structure within a single focus has not been studied with such markers. Although *T. b. rhodesiense* is genetically very closely related to *T. b. brucei* [40]–[42], it is not clear whether genetic exchange occurs in *T. b. rhodesiense* populations. The basis of human infectivity in *T. b. rhodesiense* has been understood for some time, and is due to the expression of a single gene, the serum resistance associated (*SRA*) gene [43]. By using *SRA* as a marker, the detection of *T. b. rhodesiense* parasites in non-human hosts has become more straightforward [34], [44], [45]. The genotyping of parasites isolated from foci of human disease have led to the conclusion that *T. b. rhodesiense* is clonal [10], [46], suggesting that a few parasite genotypes carrying the *SRA* gene amplified in the human population, resulting in an epidemic clonal expansion. However, these genotypes were also stable over time [10], suggesting that *T. b. rhodesiense* was not mating with the genetically more diverse sympatric *T. b. brucei* population, within which evidence for frequent mating was demonstrated. However it is clear that, unlike *T. b. gambiense* group 1, there do not seem to be biological barriers to *T. b. rhodesiense* mating with *T. b. brucei*, as this has been demonstrated in the laboratory in two separate crosses with different *T. b. brucei* strains [47], [48]. The disparity between laboratory and field data suggests that it is important to analyse further foci of *T. b. rhodesiense* and so examine populations in different epidemiological settings in order to rigorously address the question of clonality in this human infective sub-species. This will also allow a series of questions to be addressed, such as whether *T. b. rhodesiense* HAT foci in different geographical regions display similar levels of clonality; whether different foci are genetically distinct from each other, as well as from local *T. b. brucei* populations; and whether clonal populations of *T. b. rhodesiense* are stable over space and time.

To clarify our understanding of *T. b. rhodesiense* populations, we have employed microsatellite markers to determine allelic variation and multilocus genotypes from parasites isolated from three different foci of disease in East Africa, two in Uganda, and one in Malawi. The microsatellite loci were selected from a panel of genome wide markers, which had been used in the construction of the first genetic map of the parasite [16]. We have avoided ascertainment bias by employing a whole genome amplification technique on bloodspots taken directly from infected individuals [49] for all samples collected after 2001, allowing direct assessment of parasite populations by multilocus genotyping. These tools and approaches will allow us to address the following questions; (1) are different foci of *T. b. rhodesiense* genetically distinct? (2) Are the population structures and the role of genetic exchange similar in different foci? (3) By analysing samples over a period of 45 years from in and around the clonal Tororo focus, are the multilocus genotypes stable over time?

## Materials and Methods

### Ethics statement

This study was conducted according to the principles expressed in the Declaration of Helsinki. All patients recruited received written and verbal information explaining the purpose of this study and gave informed written consent. All protocols were approved by ethics committees in Uganda (Uganda Ministry of Health) and Malawi (Malawi College of Medicine) as appropriate. Furthermore, the protocols, information forms and consent forms were reviewed and approved by the Grampian Research Ethics Committee (Aberdeen, UK). Ethical consent forms were designed in English and also translated into local languages. Consent was given as a signature or a thumb print after verbal explanation. For those under 16 years of age consent was given by their legal guardian, and for those whose clinical condition prohibited full understanding of the recruitment process, consent was gained from a spouse or other family member.

### Study sites and subjects

HAT patients presenting to local hospitals or identified during community surveillance were recruited in South-Eastern Uganda in 2002 and 2003 from an extensive focus of *T. b. rhodesiense* transmission covering the Tororo, Iganga, Jinja and Busia districts [50]. This will be referred to henceforth as the Tororo focus. The second focus sampled was Soroti, where HAT emerged as a new epidemic in 1998/1999 [51], which was sampled in 2003. During this study we examined 30 samples from the Tororo focus and 88 from the Soroti outbreak. These samples were compared with 52 previously isolated and described samples (from both humans and cattle) collected from the Ugandan/Kenyan border region (including Tororo, Busia, Iganga and Jinja districts in Uganda, and Busia and Nyanza districts in Kenya), covering a period of 36 years (1961 to 1997) prior to the more recent outbreaks in Tororo and Soroti. This set of samples will be referred to as ‘Ug/Ke 61–97’ (for sample details see Table S1). These samples provide a representative snapshot of the wider geographic focus for the decades prior to 2003, and provide a useful reference point as they have previously been described as a temporally stable clonal complex [46]. This will allow us to investigate genetic links and population stability between the 2003 Ugandan outbreaks and the historical *T. b. rhodesiense* population. Samples were identified as being *T. b. rhodesiense* if they were isolated from an HAT patient or if they were able to resist the lytic effects of human serum [10], [46].

Twenty eight patients were sampled from the Central Malawi HAT focus and were recruited after admission to Nkhotakota General Hospital between 2002 and 2003. Suspect cases were initially identified by clinical surveillance teams in communities within and on the periphery of the Nkhotakota Wildlife Reserve.

Patient recruitment protocol has been previously described in [52]. Briefly, diagnosis of infection was by microscopic detection of trypanosomes in wet blood films, Giemsa stained thick blood films or in the buffy coat fraction after microhaematocrit centrifugation. Blood was collected by venipuncture from consenting patients, and collected as either 1 ml samples or as 200 µl spots on FTA filter (Whatman) cards. Samples from the Ug/Ke 61–97 focus were grown in mice and have previously been described [46]. A full list of all samples and their geographic and temporal origin is available in Table S1.

### Sample preparation

For samples isolated on FTA cards, discs of 2 mm diameter were cut from each blood spot using a Harris Micro-punch (Whatman). The discs were washed three times with 200 µl FTA purification reagent (Whatman), and twice with 200 µl 1 mM TE buffer pH 8.0, with incubation for 5 minutes at each wash. The washed discs were then used as substrate for multiple displacement amplification (MDA) whole genome amplification reactions. Whole genome amplification was carried out using the GenomiPhi DNA Amplification kit (Amersham) as described previously [49]. Three independent reactions were carried out for each sample and the reaction products pooled. Where whole blood samples were available DNA was prepared from 1 ml of blood using the Qiagen DNA blood mini kit, following the manufacturer's protocol. MDA products and DNA samples were routinely stored at −20°C prior to use.

### Polymerase Chain Reaction (PCR)-based genotyping

One µl of each MDA product or purified DNA was used as PCR template in a volume of 10 µl. The seven microsatellite loci (Ch1/18, Ch2/PLC, Ch3/5L5, Ch3/IJ15/1, Ch4/M12C12, Ch5/JS2 and Ch9/4) have been described previously [16]. Markers Ch3/5L5 and Ch3/IJ15/1, although both on chromosome 3, are 1.2 Mb apart and effectively unlinked [16]. Oligonucleotide primers (both primary and nested) for each marker are detailed in Table S2. PCR conditions were: PCR buffer (45 mMTris-HCl pH 8.8, 11 mM (NH_4_)_2_SO_4_, 4.5 mM MgCl_2_, 6.7 mM 2-mercaptoethanol, 4.4 µM EDTA, 113 µg.ml^−1^ BSA, 1 mM of each four deoxyribonucleotide triphosphates), 1 µM of each oligonucleotide primer, and 1 unit of Taq polymerase (Abgene) per 10 µl reaction. For nested reactions, 1 µl of a 1/100 dilution of first round product was used as template in the second round PCR. Microsatellite PCR products were resolved by electrophoresis on a 3% Nusieve GTG agarose gel (Cambrex), and gels were stained with 0.2 µg/ml ethidium bromide and visualised under UV light.

### Allele size determination

One primer of each pair for the microsatellite nested PCR included a 5′ FAM or HEX modification, allowing size separation of products using a capillary-based sequencer (ABI 3100 Genetic Analyser; Applied Biosystems). A set of ROX-labelled size standards (GS400 markers; Applied Biosystems; Dundee Sequencing Service http://www.dnaseq.co.uk/) was included in the run, allowing accurate determination of DNA fragment size. Data were analysed using Peak Scanner v1.0 software (Applied Biosystems). A multilocus genotype (MLG) for each isolate was defined by the specific combination of alleles across the seven loci (Table S1). Genotypes were defined as heterozygous at a marker if two peaks were detected, whereas homozygotes were represented by a single peak. Mixed infections were defined by the presence of more than two alleles for any one marker.

### Genetic analysis

Analysis of MLGs used Clustering Calculator (http://www2.biology.ualberta.ca/jbrzusto/cluster.php) generating a Phylip Drawtree string (unweighted arithmetic average clustering method, and Jaccard's similarity coefficient), which was converted into a dendrogram by Treeview (http://taxonomy.zoology.gla.ac.uk/rod/treeview.html) [53], with the dendrogram colour coded according to sample origin. Clustering Calculator generated the bootstrap values for dendrograms, using 100 iterations. Marker polymorphism and heterozygosity, Nei's genetic distance (D) and Wright's fixation index (F_ST_) between sample populations, were calculated using GenAlex [54]. Principal Component Analysis (PCA) of the MLGs was performed in GenAlEx following determination of genetic distance with data standardisation. Linkage disequilibrium between paired loci was examined using GDA. eBURST software (http://eburst.mlst.net/default.asp) was used to analyse the clonal expansion of the Ugandan genotypes and identify putative ‘founder’ genotypes [55]. The most stringent setting was used for analysis, in which isolates assigned to the same group are single locus variants (SLV; 6/7 identical loci). In order to use this software, genotypes were treated as described by Stevens and Tibayrenc [28], whereby different combinations of alleles at each locus (for example homozygotes and heterozygotes that share a common allele) are treated as distinct alleles.

## Results

### Genotypic diversity

One hundred and ninety-eight infected blood samples were examined from three distinct, active HAT foci in 2003, two in South-Eastern Uganda (‘Tororo’ and ‘Soroti’), and one in Malawi. In addition, 52 samples from the Tororo focus were collected between 1961 and 1997 (referred to as‘Ug/Ke 61–97’) and include 28 samples collected in the period 1988–90. During the period between 1990 and 2003, the Tororo focus is considered to have seeded the outbreak in Soroti, which has been linked to the restocking of cattle herds in the region [51]. The shared lineage of the Ugandan samples thus comprises a unique case study, allowing us to examine both the progress of a continuous endemic focus (Tororo) and the establishment of a new, but linked, focus (Soroti). The final focus in Malawi is endemic and still active. However, in contrast to the relatively severe and acute disease observed in Uganda, Malawian HAT is characterised as a chronic disease with slower progression to the late (meningoencephalitic) stage [50], [52], [56]. Thus the Malawi *T. b. rhodesiense* focus can be distinguished from those in Uganda by both pathogenesis and geography, but they have not been compared genetically.

Comparative analysis of these four populations, therefore, allows us to rigorously examine the role of both space and time in shaping the population dynamics of *T. b. rhodesiense*. This has been achieved through the use of seven previously described single-locus microsatellite markers, which have been physically and genetically mapped to six different megabase chromosomes of *T. brucei*. Of the 198 samples, full multilocus genotypes (MLGs) were obtained for 176, with the remainder genotyped for at least four of the seven loci (Table S2). Three samples, one from Tororo (LIRI017) and two from Ug/Ke 61–97 (K237 and UgE90) were identified as mixed genotypes by the presence of three microsatellite alleles for at least one of the seven loci and have therefore been excluded from further analysis (data not shown). A summary of the basic population genetic features of each of the four populations, based on the MLGs, is presented in Table 1. The population from Malawi clearly differs from the Ugandan populations in that the number of distinct MLGs approaches the number of samples whereas the proportion of distinct MLGs is much lower in the three sets of Ugandan samples. This difference is further emphasised by the observed and expected heterozygosities and the values of the fixation index. Thus the Malawi population shows much higher levels of diversity than those from Uganda.

**Table 1:** Basic population genetic parameters of the four *T. b. rhodesiense* populations.

Population	n	P	A	He	Ho	F_IS_
Ug/Ke 61–97	43/21	0.86/0.86	4.00/4.00	0.35/0.42	0.40/0.43	−0.15/−0.03
Tororo	26/17	1.00/1.00	3.29/3.00	0.46/0.47	0.71/0.67	−0.56/−0.44
Soroti	84/18	0.86/1.00	3.14/3.14	0.32/0.39	0.58/0.60	−0.81/−0.56
Malawi	23/20	1.00/1.00	3.00/3.00	0.42/0.42	0.40/0.39	0.06/0.06

n = ‘all samples/unique MLGs (n)’, respectively, p = proportion of polymorphic loci, A = mean allele number per locus, He = Expected heterozygosity, Ho = Observed heterozygosity, F_IS_ = fixation index; the first number in each cell is measurement with all samples, the second number is after removal of repeated genotypes.

### *T. b. rhodesiense* is sub-structured by geography

**Figure 1:**
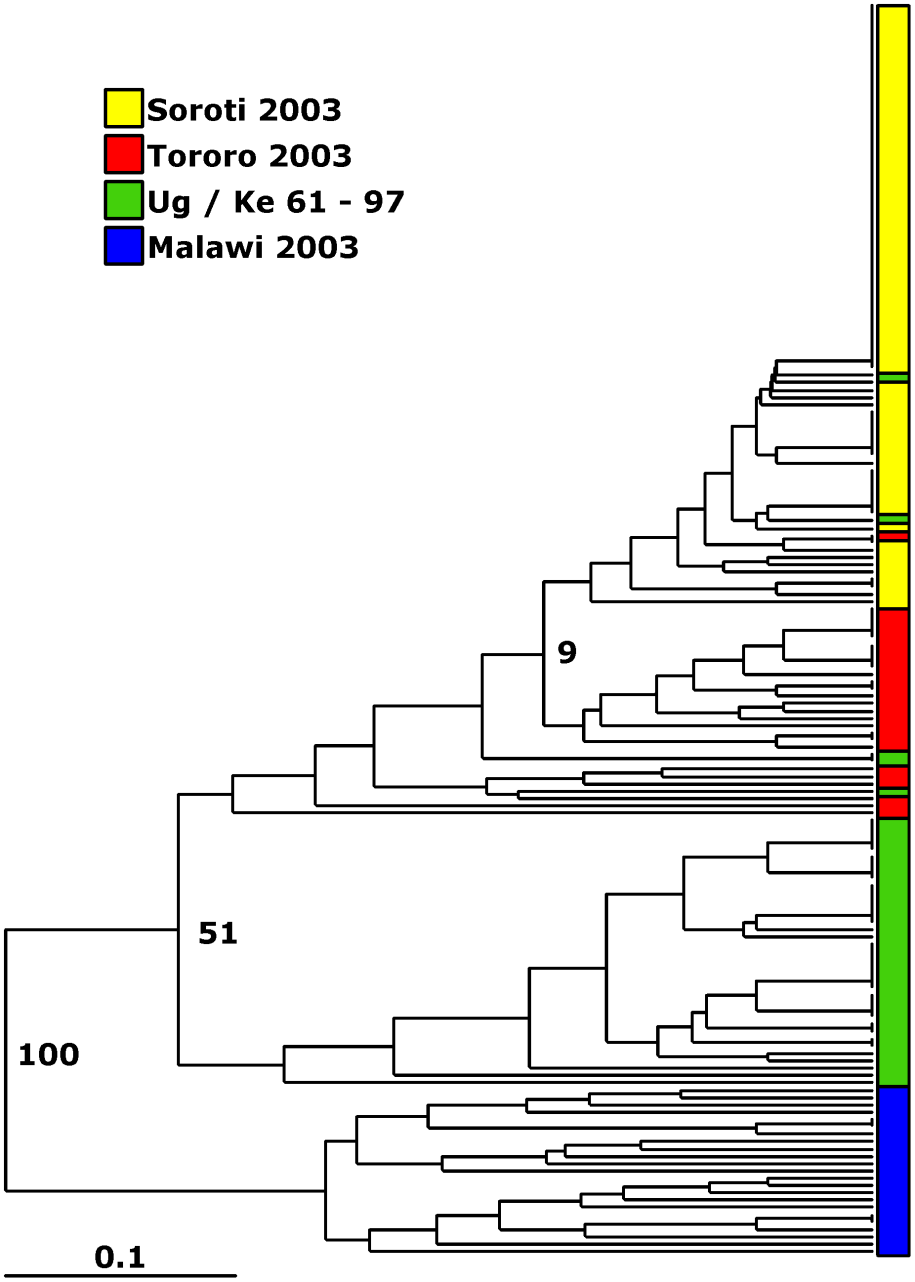
Neighbour joining tree of isolates included in study, constructed using Nei's genetic distance. Significant separation of the Malawi population from those in Uganda is shown (bootstrap values are labelled for significant nodes) while within Uganda the three populations cannot be significantly resolved. Populations: Malawi = blue, Ug/Ke 61–97 = green, Soroti = yellow, Tororo = red.

**Figure 2:**
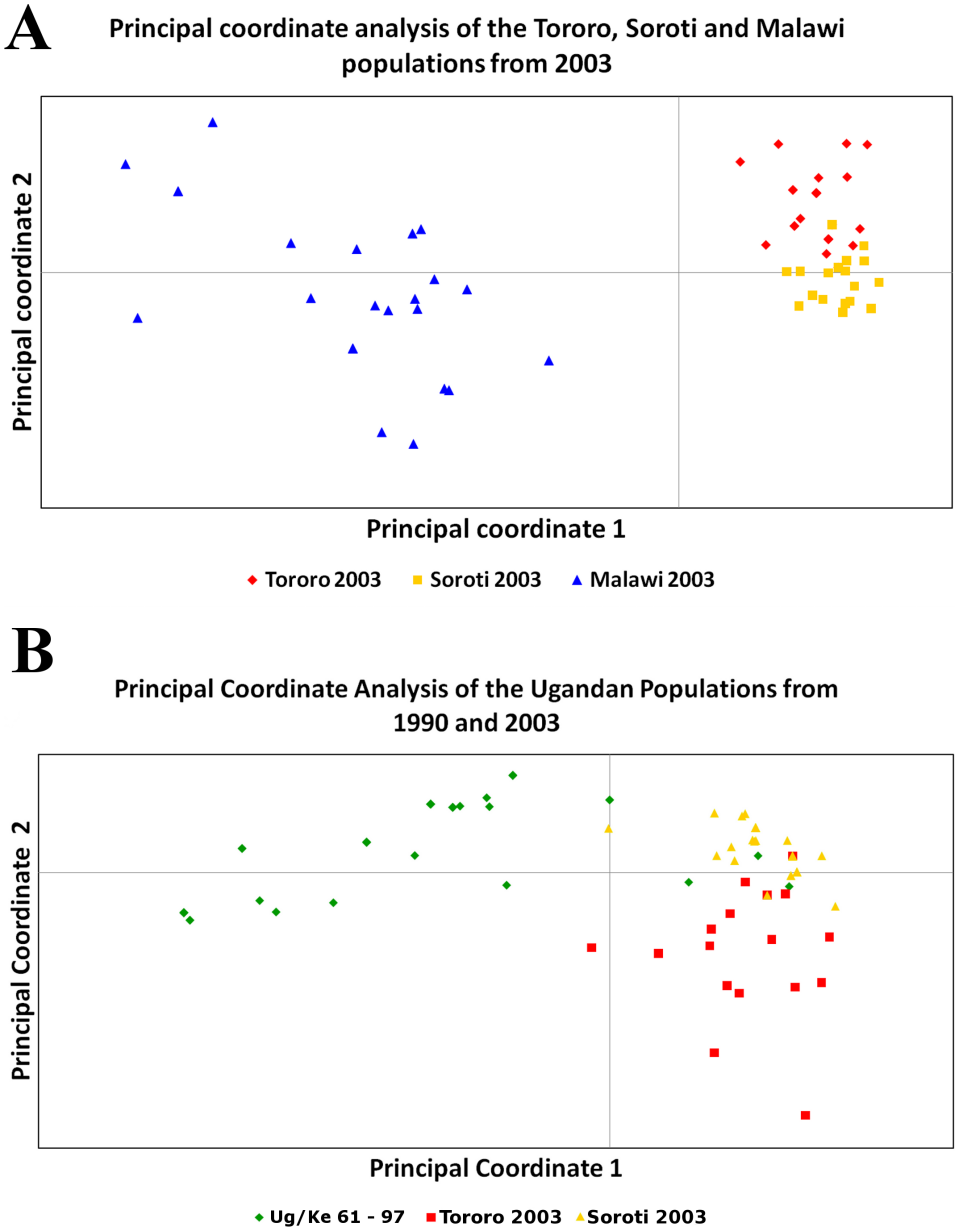
A. Principle Component Analysis of isolates collected in 2003. Coordinate 1 accounts for 70% of the variation observed and separates the Malawi population from those in Uganda. Principal coordinate 2 accounts for 12% of the total variation, partially separating the two Ugandan populations, in addition to highlighting the diversity within Malawi. B. Principal Component Analysis of the isolates collected in Uganda. Coordinate 1 accounts for 58% of the observed variation and separates the majority of the Ug/Ke 61–97 isolates from those collected in 2003. Principal coordinate 2 accounts for 18% of the variation and partially separates the Tororo and Soroti isolates collected in 2003. While principal coordinates 1 and 2 account for 76% of the observed variation within the sample set the three populations are not completely separated.

In order to determine if the *T. b. rhodesiense* population in East Africa was sub-structured due to geographical separation, we compared only those populations that were collected at the same time (2003), to avoid possible temporal sub-structuring. There were more private alleles in the Malawi population (eight) compared to three in Tororo and three in Soroti. Of the private alleles in Malawi five were present at frequencies above 0.1 within the population, whereas only one was above this frequency in Tororo and none in Soroti (Table S3). Nei's unbiased genetic distance (D) and pairwise population F_ST_ were measured, indicating that the Ugandan populations are closely related, although the Soroti and Tororo populations are more closely related to each other than either is to the population from Tororo sampled from 1961–97 (Table 2). The Malawi population shows substantial genetic differentiation from the Ugandan samples by both measures (Table 2). The dendrogram of similarity (Fig. 1) confirms the significant separation of the Malawi population from those in Uganda (100% bootstrap support). The Ugandan populations could not be resolved with high confidence, although there is some support for the separation of the Ug/Ke 61–97 population from the Soroti and Tororo 2003 populations (Fig. 1). Principal Component Analysis (PCA) of the MLGs from these populations identified two co-ordinates that accounted for more than 80% of the variation (Fig. 2A). These highlight the separation of the Malawi population and the similarity within the Ugandan populations. Principal coordinate 1, accounting for 70% of observed variation, primarily separates the populations based on country of origin, while coordinate 2 (12% of the variation) partially separates the two Ugandan populations as well as highlighting the high level of diversity within the Malawi focus. The PCA plot indicates that while the genotypes from the Tororo and Soroti foci in 2003 were closely related, the populations are genetically distinct albeit with some overlap. All of these data combine to demonstrate that there is significant genetic differentiation between the Malawian and Ugandan *T. b. rhodesiense* isolates, indicating population sub-structuring due to geography.

**Table 2:** Pairwise values of Wright's fixation index (F_ST_; above diagonal) and Nei's genetic distance (*D*; below diagonal) between populations of *T. b. rhodesiense* as defined by focus and time.

	Ug/Ke 61–97	Tororo	Soroti	Malawi
Ug/Ke 61–97	-	0.201	0.203	0.267
Tororo	0.411	-	0.109	0.226
Soroti	0.345	0.129	-	0.266
Malawi	0.712	0.669	0.680	-

### The Tororo outbreak in 2003 shows no evidence for mating and is distinct from the Ug/Ke 61–97 isolates

The Ug/Ke 61–97 isolates are representative of the historical population of *T. b. rhodesiense* present in South East Uganda over a period of 36 years with a significant sample set from 1988/90 [57]. The availability of these historical isolates allowed us to analyse if the trypanosomes had remained genetically stable over time or if new genotypes have appeared, for example by migration or mutation. Twenty-six samples collected from Tororo in 2003 were fully genotyped including one mixed infection identified (LIRI017), which was removed from the analysis, together with a further three partially genotyped samples. Samples from 52 individuals from the Ug/Ke 61–97 sample set were genotyped. Two contained multiple infections and were removed from this study, while 43 of the remaining 50 were fully genotyped for seven microsatellite markers. As these samples were from several geographic locations within the focus (Busia, Busoga and Nyanza – encompassing an area of ∼100 km from Tororo) and collected over several decades we did not attempt to examine this population for indices of mating, to avoid errors due to temporal or geographical sub-structuring. Analysis showed that the dominant MLGs identified in Ug/Ke 61–97 and Tororo2003 were distinct. In Ug/Ke 61–97 the dominant MLGs were MLG 65, 69 and 75, whereas in 2003 the dominant MLGs were MLG 24 and 27 (Tables 3 & S1). One of the most striking differences occurs at locus Ch4/M12C12, which was completely monomorphic in the Ug/Ke 61–97 population. By 2003, three additional alleles had arisen within the population to the point that the predominant allele from Ug/Ke 61–97 was present at a frequency of 0.53, largely as part of a heterozygote pair that dominates the Tororo2003 population (Tables S1 & S3). Additionally, examination of the genetic distance between the populations using Nei's unbiased genetic distance (D) and F_ST_, indicates that while the two populations are highly related they can be distinguished using these measures (Table 2).

**Table 3:** Probability of agreement with Hardy Weinberg predictions (data shown for ‘all samples/unique MLGs’, respectively).

	Tororo	Soroti	Malawi
Ch3/5L5	**0.00/**0.06	**0.00/0.00**	0.29/0.29
Ch4/M12C12	**0.00/0.00**	1.00/1.00	**0.00/**0.05
Ch2/PLC	1.00/1.00	**0.00/0.03**	1.00/1.00
Ch5/JS2	0.08/0.64	1.00/1.00	**0.00/0.00**
Ch1/18	**0.01/**0.11	**0.00/0.01**	1.00/1.00
Ch9/4	**0.00/0.00**	**0.00/0.04**	**0.02/0.04**
Ch3/IJ15/1	**0.00/0.04**	1.00/1.00	0.06/0.15

In terms of the population structure, an excess of heterozygotes at six out of seven loci was observed in the Tororo2003 samples, while five of the seven loci displayed significant deviation from Hardy-Weinberg predictions, indicating a departure from panmixia (Table 4). However the two markers that displayed agreement with Hardy-Weinberg predictions, Ch2/PLC and Ch5/JS2, had low polymorphism (Table S3) and so could be susceptible to Type 2 error. When duplicate genotypes were removed, two additional markers, Ch1/18 and Ch3/5L5 show agreement with Hardy-Weinberg predictions (Table 4). However, after removal of the repeated MLGs, only 17 individuals remain in the population. Examining the genotypes from this population at each locus, it is clear that six of the loci are predominantly heterozygous for two alleles while the remaining locus is largely homozygous (Table S1) and this genotypic structure precludes any meaningful analysis of linkage disequilibrium. This, coupled with the occurrence of four MLGs (Table 3) that are found multiple times (accounting for 50% of the population), is suggestive of little or no sexual recombination.

**Table 4:** Linkage equilibrium/disequilibrium in *T. b. rhodesiense* populations and the frequency of repeated genotypes.

Population	Sample size	Pairs of loci in LD (all)	Pairs of loci in LD (unique)	Repeated MLGs (number)
Ug/Ke 61–97	43	12/15	8/15	65 (6), 69 (6); 75 (5); 67 (4); 73 (4); 71 (2); 68 (2)
Malawi	23	2/21	1/21	1 (2); 5 (2)
Tororo	26	nd	nd	27 (4); 24 (5), 57(2)
Soroti	84	nd	nd	49 (50); 42 (7); 31 (7); 21 (2)

nd = not done, as analysis not appropriate.

**Figure 3:**
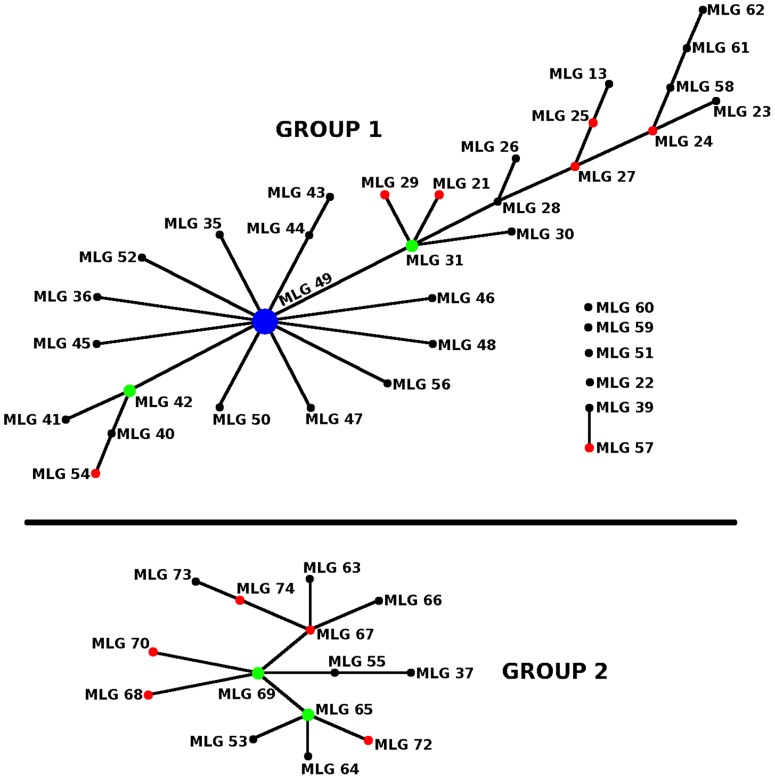
eBURST analysis of the Ugandan samples. The putative founder genotype (SER002) is at the centre of the star-shaped radial lineage. Each node differs from its immediate neighbour by a single locus (i.e. the isolates are identical to each other at 6/7 loci), and is labelled with a representative isolate name.

The data from Tororo in 2003 suggest little or no mating due to the presence of multiple dominant repeated genotypes and significant disagreement from Hardy-Weinberg expectations at the majority of loci. The data also suggests that the genotypes present in the Ug/Ke 61–97 and Tororo 2003 populations are different. Analysis by PCA of the Ugandan populations (Fig. 3B) provides further evidence for this conclusion with the Ug/Ke 61–97 and Tororo 2003 populations clustering separately.

### The population of the Soroti focus in 2003 is genetically homogeneous, consistent with a founder effect

The Soroti focus, unlike those of Tororo and Malawi, is relatively new as human cases of trypanosomiasis in this district were first reported in 1998. The focus has since been identified as an offshoot of the Tororo epidemic [51]. Subsequent implementation of disease control measures including tsetse trapping and treatment of livestock have been unable to contain the outbreak, with over 400 cases reported between 1998 and 2004 [58]. Fitting with the suggested origins of this disease focus, the population sampled is most closely related (by measurement of Nei's genetic distance and F_ST_) to that of Tororo 2003 (Table 2). While the Soroti population represents the largest sample size, with 84 individuals fully genotyped, the majority of these represent replicate MLGs (Table 3) as only 18 complete and unique MLGs were identified. The most frequent repeated genotype is MLG 49, which is represented 50 times in total. The presence of many parasites with the same genotype constituting more than 59% of the population clearly demonstrates that this population is not panmictic.

Comparison of the genotypes identified in the Soroti population with those from the two Tororo populations using similarity analysis (Fig. 1) shows that they are closely related. While members of each population broadly cluster together but separately from the Malawi population and, with less convincing bootstrap support, the Ug/Ke 61–97 population, there is limited bootstrap support for the Ugandan clusters. However, PCA analysis of the MLGs (Fig. 2B) clearly shows that Soroti and Tororo (2003) populations are closely related to each other but both are more distinct from the Ug/Ke 61–97 population - the two co-ordinates account for 76% of the diversity within this dataset. Furthermore, the relative tightness of the clusters of genotypes from each population reflects the level of diversity within each, with Ug/Ke 61–97 showing a broader scatter reflecting its higher level of diversity. The most frequent MLG in the Soroti population (MLG 49) is not observed in the Tororo 2003 population but the two populations share MLGs 29 and 31 with the latter occurring once in Tororo but seven times in Soroti (Table 3), suggesting the possibility that it might have been a founder genotype in Soroti. To explore the genetic relationships between the genotypes from Soroti and the two Tororo populations and so provide insight into the origins of the Soroti outbreak, the genotypes were analysed using eBURST (Fig. 3). The analysis defines two distinct groups of genotypes one (Group 1) comprising mostly the Soroti and Tororo 2003 isolates (albeit with two MLGs from the Ug/Ke 61–97 population; MLGs 40 and 54), and the second (Group 2) comprising the bulk of the Ug/Ke 61–97 isolates. These results indicate a direct genetic lineage of the Soroti isolates deriving from the Tororo 2003 isolates, consistent with the proposed import into Soroti from Tororo [51]. The predominance of a single clone in Soroti suggests that the import has occurred relatively recently, and probably involved very few MLGs from Tororo, as evidenced by the clonal nature of the Soroti complex, which shows very little genetic divergence in comparison with the more longstanding outbreak in Tororo, where genetic changes have accumulated over time. Group 2 is composed of fewer closely related single-locus variants, resulting from the greater diversity in the Ug/Ke 61–97 population seen by other measures. This may be a reflection of the fact that the number of cases was relatively high in the 1980s into 1990, but then dramatically decreased through the 1990s [59], and this significant reduction in cases and therefore *T. b. rhodesiense* population offers a potential explanation for the bottleneck effect of the subsequent emergence of a very few surviving genotypes that founded the outbreaks seen in 2003 and onwards.

This, in addition to the close relationship to the 2003 Tororo focus, is consistent with the data that Soroti represents an off-shoot population and suggests the population has been through a recent bottleneck, based on the establishment of a population by a limited number of founder individuals.

### The population structure of the Malawi focus in 2003 indicates frequent mating

The Malawi population, genetically distinct from those in Uganda (Fig. 1 and Table 2), comprises 28 individuals, with 23 fully genotyped with all seven markers. Twenty-one of the 23 MLGs observed are unique within the population. Examination of the markers for agreement with Hardy-Weinberg expectations revealed three loci, Ch4/M12C12, Ch5/JS2 and Ch9/4 that deviate significantly from predictions (Table 4). Disagreement at Ch4/M12C12 and Ch5/JS2 results from heterozygote and homozygote excesses, respectively. For marker Ch9/4 the disagreement arises from the presence of a single individual homozygous for a rare allele. Among the markers both Ch2/PLC and Ch1/18 are dominated by single alleles within the population (Table S3), possibly accounting for the complete agreement at these loci (Type 2 error). While only two repeated genotypes were observed their removal from the population results in Ch4/M12C12 moving to agreement with Hardy-Weinberg. Analysis of the combinations of alleles at pairs of loci showed that only 2 out of 21 loci combinations showed significant evidence of linkage disequilibrium (Tables 3 & S4), which is reduced to a single locus combination (Ch9/4 – Ch3/IJ15/1) once repeated genotypes were removed. The high proportion of unique genotypes observed within this population, coupled with agreement with Hardy-Weinberg and lack of linkage disequilibrium is consistent with the occurrence of a level of recombination within the population. Additionally, the F-statistics for this population suggest that there is an appreciable degree of mating occurring, as the value is close to zero (Table 1), in contrast to the Ugandan populations, where there is significant deviation from zero. Although the number of samples is relatively low (23) and we therefore cannot robustly conclude that the population is panmictic, additional evidence is provided by the fact that the genetic diversity observed within the Malawi cohort is much greater than that in the Ugandan samples (Fig. 1 and Fig. 2A). In summary, the Malawi focus is genetically diverse, displays allelic segregation in the population and there is limited LD consistent with frequent mating. This is the first time that this has been observed for *T. b. rhodesiense* in the field.

## Discussion

Our results provide evidence that the causative agent of East African Sleeping Sickness, *T. b. rhodesiense*, can undergo genetic exchange in the field in Malawi, in contrast to previous studies that have described *T. b. rhodesiense* as a genetically homogeneous variant of *T. b. brucei*. Unlike the situation in Malawi, the Ugandan populations analysed provided no evidence for the occurrence of frequent genetic exchange and conform with the accepted concept of *T. b. rhodesiense* as a related set of stable clones in the two foci of disease in Uganda. Thus, the population structure and the role of genetic exchange within this sub-species differs in different geographical regions making it difficult to draw general conclusions about the sub-species as a whole, and so questions the description of *T. b. rhodesiense* as a genetically homogeneous human infective variant of *T. b. brucei*.

One question that these findings raise is why mating occurs in the Malawi focus but not in the Ugandan foci. The available laboratory data show that mating can occur between *T. b. rhodesiense* and *T. b. brucei*, albeit using a Zambian human infective isolate, and so show that *T. b. rhodesiense* has the ability to undergo genetic exchange [47], [48]. In Uganda, it is known that both *T. b. brucei* and *T. b. rhodesiense* are prevalent in non-human mammalian hosts, notably livestock [10], [34], [46], and are therefore likely to be cycled through the tsetse fly together, providing the opportunity for genetic exchange, particularly as *T. b. brucei* undergoes genetic exchange itself. In this scenario, one would predict that *T. b. rhodesiense* would undergo genetic exchange, show high levels of diversity and not be distinguishable from *T. b. brucei* except by the presence of the *SRA* gene. The available evidence does not support this as firstly we have shown (in Soroti and Tororo) that the populations are of low diversity with frequent identical genotypes and secondly previous studies have shown that *T. b. brucei* can be distinguished from *T. b. rhodesiense* by RFLP and minisatellite markers [10], [60], demonstrating that they are genetically isolated. Based on these considerations, one hypothesis to explain the results is that Ugandan *T. b. rhodesiense* has lost the ability to undergo genetic exchange. This could be tested by attempting laboratory crosses with these strains. In contrast, our data support the occurrence of genetic exchange in Malawian *T. b. rhodesiense* and so one would predict that genetic exchange would also occur with local *T. b. brucei* with human infection occurring when the *SRA* gene is inherited. Unfortunately no viable Malawian *T. b. brucei* strains are available and so it is not currently possible to test this hypothesis.

The genotyping of isolates from the two foci in Uganda not only provides important information about the role of genetic exchange in these populations but also information about the temporal genotypic stability in Tororo and the potential origin of the Soroti outbreak. Our data show that genetic exchange is limited or does not occur in these populations based on the lack of agreement with Hardy-Weinberg predictions, high levels of heterozygosity, linkage disequilibrium and the high frequency of identical genotypes. These findings lead to the conclusion that these populations are clonal, primarily evolving by mitotic division and mutation. This conclusion agrees with previous analysis of the Ug/Ke 61–97 population using minisatellite markers [10] where two predominant genotypes represented much of the population and, furthermore, these were stable over time based on the analysis of a few isolates from 1961 [10]. Our data presented here provide a higher resolution analysis by using a larger number of markers and provide a further test of the stability of clonal trypanosome populations in space and time. The genotypic comparison between Ug/Ke 61–97 and Tororo 2003 provides a novel finding that stability over time may not be a feature of these populations. Using similarity analysis (Fig. 1), PCA (Fig. 2B) and eBURST (Fig. 3), the two populations are different – although they do share a small number of common MLGs, the dominant MLGs are different. The two populations show similarity in that they both contain multiple repeated genotypes as well as a number of common alleles (Tables S2 & S3). However, the eBURST analysis separates the two populations into distinct, but related, clusters. As the two populations were sampled 13 years apart and there is no evidence for genetic exchange, we must assume that either mutation accounts for these differences and has occurred at several loci over this time span, or alternatively there has been a degree of migration and introduction of some novel genotypes. This is in marked contrast to the similarities between the Soroti and Tororo 2003 populations, which are highly related by PCA and similarity analysis (Fig. 1 and 2) as well as sharing two MLGs (MLG 29 and 31). The predominant MLG in Soroti (MLG 49) is, however, not observed in Tororo but the eBURST analysis (Fig. 3) shows that this MLG differs by a single allele from a series of the other MLGs in the population by a classical star like relationship characteristic of a clonal population. MLG 49 differs by a single allele from MLG 31 (present in both populations), which occurs seven times in the Soroti population and is related to MLG 29 (also present in both populations) by a further single allelic difference. Based on these data, a hypothesis for the origin of the Soroti focus is that it was seeded by MLGs 31 and 29 from Tororo, which mutated to generate MLG 49 and subsequently the other related genotypes. As Tororo was not sampled at the time point when the cattle were moved into Soroti and initiated the outbreak, this hypothesis cannot be tested directly. However the genotype data add strong support to the conclusions reached by Fevre et al. 2001 [51] as to the origin of the Soroti outbreak. Even though the two populations are very similar and do not undergo significant levels of recombination, it is again clear that the genotypes are not wholly stable in time and place but form a clonal complex often dominated by a single or a few highly related genotypes.

These findings have implications for our understanding of recombination as an evolutionary driving force in trypanosomes. It is clear that mating plays different roles in different species, with *T. vivax* and *T. b. gambiense* being clonal [36], [37], [61], whereas *T. b. brucei* and *T. congolense* can undergo frequent mating [10], [62]. However, *T. b. rhodesiense* provides evidence for these differences being displayed within a sub-species. The identity of the trigger for whether mating occurs or not within these species or subspecies is obviously a key question to address, but it seems reasonable to assume that it is likely to depend upon certain epidemiological scenarios (e.g. transmission intensity, reservoir host population, tsetse species etc). This plasticity in the use of sexual recombination within a genus, and particularly within a species (*T. b. rhodesiense* versus *T. b. brucei* presenting a prime example), makes trypanosomes a unique paradigm for studying the evolution of sexual recombination, and the role that mating plays in shaping the responses to epidemiological selective pressures.

## Supporting Information

Table S1 Sample origin and multi locus genotype (MLG) data for the 195 single genotype samples. Genotype data lists allele size in base pairs with missing data represented by 0. MLG IDs have not been assigned to samples with missing data. * This MLG was observed in both the Soroti and Tororo populations.
(DOCX)

Table S2 Microsatellite loci and the primers used for their amplification. For each locus the first pair of primers were used for the primary reaction and the second pair for the subsequent nested reaction.
(DOCX)

Table S3 Allele frequencies for the seven microsatellite markers in all four trypanosome populations.
(DOCX)

Table S4 Linkage disequilibrium between pairs of loci for the two populations where analysis was warranted for ‘all samples/unique MLGs’, respectively. Allele combinations were preserved for loci showing significant disagreement with HWE predictions. *P<0.05 = Significant linkage disequilibrium, indicated in bold.
(DOCX)
