# Supplementary material for: Population Genetics of Trypanosoma brucei rhodesiense: Clonality and Diversity within and between Foci
Source: PLoS Negl Trop Dis. 2013 Nov 14;7(11):e2526. doi: 10.1371/journal.pntd.0002526 (PMC3828156; doi:10.1371/journal.pntd.0002526)
Supplement: Table S1 — Sample origin and multi locus genotype (MLG) data for the 195 single genotype samples. Genotype data lists allele size in base pairs with missing data represented by 0. MLG IDs have not been assigned to samples with missing data. * This MLG was observed in both the Soroti and Tororo populations. (DOCX) [file pntd.0002526.s001.docx]

Supporting Information Table 1. Sample origin and multi locus genotype (MLG) data for the 195 single genotype samples. Genotype data lists allele size in base pairs with missing data represented by 0. MLG IDs have not been assigned to samples with missing data. * This MLG was observed in both the Soroti and Tororo populations

| Sample | Population | Focus of origin | Collection period | Ch1/18 | | Ch2/PLC | | Ch3/IJ15/I | | Ch3/5L5 | | Ch4/M12C12 | | Ch5/JS2 | | Ch9/4 | | MLG |
| --- | --- | --- | --- | --- | --- | --- | --- | --- | --- | --- | --- | --- | --- | --- | --- | --- | --- | --- |
| NKK/T/022 | Malawi | Malawi | 2002-2003 | 153 | 162 | 136 | 136 | 132 | 145 | 111 | 111 | 98 | 107 | 98 | 98 | 140 | 140 | 1 |
| NKK/T/025 | Malawi | Malawi | 2002-2003 | 153 | 162 | 136 | 136 | 132 | 145 | 111 | 111 | 98 | 107 | 98 | 98 | 140 | 140 | 1 |
| NKK/T/042 | Malawi | Malawi | 2002-2003 | 162 | 162 | 136 | 136 | 145 | 145 | 111 | 111 | 98 | 107 | 98 | 98 | 148 | 148 | 2 |
| NKK/T/037 | Malawi | Malawi | 2002-2003 | 162 | 162 | 136 | 136 | 120 | 120 | 111 | 111 | 98 | 107 | 169 | 169 | 140 | 148 | 4 |
| NKK/T/030 | Malawi | Malawi | 2002-2003 | 162 | 162 | 136 | 136 | 120 | 145 | 111 | 111 | 98 | 107 | 169 | 169 | 140 | 148 | 5 |
| NKK/T/058 | Malawi | Malawi | 2002-2003 | 162 | 162 | 136 | 136 | 120 | 145 | 111 | 111 | 98 | 107 | 169 | 169 | 140 | 148 | 5 |
| NKK/T/054 | Malawi | Malawi | 2002-2003 | 162 | 162 | 136 | 143 | 115 | 145 | 111 | 111 | 98 | 107 | 98 | 98 | 148 | 148 | 6 |
| NKK/T/053 | Malawi | Malawi | 2002-2003 | 162 | 162 | 136 | 148 | 145 | 145 | 111 | 111 | 98 | 107 | 98 | 98 | 140 | 148 | 7 |
| NKK/T/006 | Malawi | Malawi | 2002-2003 | 162 | 162 | 136 | 148 | 132 | 132 | 111 | 111 | 98 | 107 | 98 | 98 | 148 | 148 | 8 |
| NKK/T/021 | Malawi | Malawi | 2002-2003 | 153 | 162 | 136 | 124 | 132 | 132 | 111 | 111 | 107 | 107 | 98 | 98 | 140 | 140 | 9 |
| NKK/T/010 | Malawi | Malawi | 2002-2003 | 153 | 162 | 136 | 136 | 132 | 145 | 111 | 111 | 107 | 107 | 98 | 98 | 140 | 140 | 10 |
| NKK/T/005 | Malawi | Malawi | 2002-2003 | 162 | 162 | 136 | 136 | 132 | 145 | 111 | 111 | 107 | 107 | 169 | 169 | 140 | 140 | 11 |
| NKK/T/044 | Malawi | Malawi | 2002-2003 | 162 | 162 | 136 | 143 | 145 | 145 | 111 | 111 | 107 | 107 | 98 | 98 | 140 | 148 | 12 |
| NKK/T/027 | Malawi | Malawi | 2002-2003 | 162 | 162 | 136 | 136 | 132 | 145 | 111 | 120 | 98 | 107 | 90 | 98 | 140 | 148 | 14 |
| NKK/T/039 | Malawi | Malawi | 2002-2003 | 153 | 162 | 136 | 136 | 115 | 145 | 111 | 120 | 98 | 107 | 98 | 98 | 140 | 148 | 15 |
| NKK/T/012 | Malawi | Malawi | 2002-2003 | 162 | 162 | 136 | 136 | 132 | 132 | 111 | 120 | 98 | 107 | 98 | 98 | 140 | 140 | 16 |
| NKK/T/035 | Malawi | Malawi | 2002-2003 | 162 | 162 | 136 | 136 | 132 | 145 | 111 | 120 | 98 | 107 | 98 | 98 | 140 | 140 | 17 |
| NKK/T/009 | Malawi | Malawi | 2002-2003 | 162 | 162 | 136 | 136 | 132 | 145 | 111 | 120 | 98 | 107 | 98 | 169 | 140 | 140 | 18 |
| NKK/T/038 | Malawi | Malawi | 2002-2003 | 162 | 162 | 136 | 136 | 115 | 132 | 111 | 120 | 98 | 107 | 98 | 169 | 140 | 148 | 19 |
| NKK/T/028 | Malawi | Malawi | 2002-2003 | 162 | 162 | 136 | 136 | 115 | 115 | 111 | 120 | 98 | 107 | 169 | 169 | 140 | 148 | 20 |
| NKK/T/007 | Malawi | Malawi | 2002-2003 | 145 | 162 | 136 | 136 | 132 | 145 | 111 | 120 | 107 | 107 | 98 | 98 | 140 | 140 | 32 |
| NKK/T/026 | Malawi | Malawi | 2002-2003 | 162 | 162 | 136 | 136 | 132 | 132 | 111 | 120 | 107 | 107 | 98 | 98 | 144 | 144 | 33 |
| NKK/T/056 | Malawi | Malawi | 2002-2003 | 162 | 162 | 136 | 136 | 115 | 145 | 111 | 120 | 107 | 107 | 169 | 169 | 140 | 148 | 34 |
| NKK/T/002 | Malawi | Malawi | 2002-2003 | 162 | 162 | 136 | 136 | 132 | 132 | 111 | 111 | 98 | 107 | 98 | 169 | 0 | 0 |  |
| NKK/T/057 | Malawi | Malawi | 2002-2003 | 162 | 162 | 136 | 136 | 0 | 0 | 111 | 120 | 107 | 107 | 98 | 98 | 0 | 0 |  |
| NKK/T/003 | Malawi | Malawi | 2002-2003 | 162 | 162 | 136 | 136 | 115 | 115 | 111 | 111 | 98 | 107 | 169 | 169 | 0 | 0 |  |
| NKK/T/049 | Malawi | Malawi | 2002-2003 | 162 | 162 | 136 | 143 | 0 | 0 | 111 | 120 | 98 | 107 | 98 | 169 | 148 | 148 |  |
| NKK/T/040 | Malawi | Malawi | 2002-2003 | 162 | 162 | 136 | 136 | 115 | 145 | 111 | 120 | 98 | 107 | 169 | 169 | 0 | 0 |  |
| SER020 | Soroti | Soroti | 2002-2003 | 162 | 176 | 136 | 151 | 141 | 141 | 111 | 120 | 98 | 107 | 98 | 98 | 144 | 161 | 21 |
| SER041 | Soroti | Soroti | 2002-2003 | 162 | 176 | 136 | 151 | 141 | 141 | 111 | 120 | 98 | 107 | 98 | 98 | 144 | 161 | 21 |
| SER062 | Soroti | Soroti | 2002-2003 | 162 | 176 | 151 | 151 | 141 | 141 | 111 | 120 | 98 | 107 | 98 | 98 | 144 | 161 | 28 |
| SER077 | Soroti | Soroti | 2002-2003 | 162 | 176 | 151 | 166 | 141 | 141 | 111 | 120 | 98 | 107 | 90 | 98 | 144 | 161 | 29* |
| SER003 | Soroti | Soroti | 2002-2003 | 162 | 176 | 151 | 166 | 141 | 141 | 111 | 120 | 98 | 107 | 98 | 98 | 133 | 161 | 30 |
| SER006 | Soroti | Soroti | 2002-2003 | 162 | 176 | 151 | 166 | 141 | 141 | 111 | 120 | 98 | 107 | 98 | 98 | 144 | 161 | 31* |
| SER007 | Soroti | Soroti | 2002-2003 | 162 | 176 | 151 | 166 | 141 | 141 | 111 | 120 | 98 | 107 | 98 | 98 | 144 | 161 | 31* |
| SER034 | Soroti | Soroti | 2002-2003 | 162 | 176 | 151 | 166 | 141 | 141 | 111 | 120 | 98 | 107 | 98 | 98 | 144 | 161 | 31* |
| SER064 | Soroti | Soroti | 2002-2003 | 162 | 176 | 151 | 166 | 141 | 141 | 111 | 120 | 98 | 107 | 98 | 98 | 144 | 161 | 31* |
| SER066 | Soroti | Soroti | 2002-2003 | 162 | 176 | 151 | 166 | 141 | 141 | 111 | 120 | 98 | 107 | 98 | 98 | 144 | 161 | 31* |
| SER067 | Soroti | Soroti | 2002-2003 | 162 | 176 | 151 | 166 | 141 | 141 | 111 | 120 | 98 | 107 | 98 | 98 | 144 | 161 | 31* |
| SER079 | Soroti | Soroti | 2002-2003 | 162 | 176 | 151 | 166 | 141 | 141 | 111 | 120 | 98 | 107 | 98 | 98 | 144 | 161 | 31* |
| SER057 | Soroti | Soroti | 2002-2003 | 162 | 176 | 136 | 151 | 141 | 141 | 111 | 120 | 107 | 107 | 98 | 98 | 144 | 161 | 35 |
| SER016 | Soroti | Soroti | 2002-2003 | 162 | 176 | 143 | 151 | 141 | 141 | 111 | 120 | 107 | 107 | 98 | 98 | 144 | 161 | 36 |
| SER001 | Soroti | Soroti | 2002-2003 | 162 | 176 | 151 | 151 | 141 | 141 | 111 | 120 | 107 | 107 | 98 | 98 | 144 | 161 | 42 |
| SER027 | Soroti | Soroti | 2002-2003 | 162 | 176 | 151 | 151 | 141 | 141 | 111 | 120 | 107 | 107 | 98 | 98 | 144 | 161 | 42 |
| SER047 | Soroti | Soroti | 2002-2003 | 162 | 176 | 151 | 151 | 141 | 141 | 111 | 120 | 107 | 107 | 98 | 98 | 144 | 161 | 42 |
| SER093 | Soroti | Soroti | 2002-2003 | 162 | 176 | 151 | 151 | 141 | 141 | 111 | 120 | 107 | 107 | 98 | 98 | 144 | 161 | 42 |
| SER098 | Soroti | Soroti | 2002-2003 | 162 | 176 | 151 | 151 | 141 | 141 | 111 | 120 | 107 | 107 | 98 | 98 | 144 | 161 | 42 |
| SER105 | Soroti | Soroti | 2002-2003 | 162 | 176 | 151 | 151 | 141 | 141 | 111 | 120 | 107 | 107 | 98 | 98 | 144 | 161 | 42 |
| SER111 | Soroti | Soroti | 2002-2003 | 162 | 176 | 151 | 151 | 141 | 141 | 111 | 120 | 107 | 107 | 98 | 98 | 144 | 161 | 42 |
| SER058 | Soroti | Soroti | 2002-2003 | 162 | 176 | 151 | 166 | 141 | 141 | 111 | 120 | 107 | 107 | 90 | 98 | 144 | 144 | 43 |
| SER042 | Soroti | Soroti | 2002-2003 | 162 | 176 | 151 | 166 | 141 | 141 | 111 | 120 | 107 | 107 | 90 | 98 | 144 | 161 | 44 |
| SER024 | Soroti | Soroti | 2002-2003 | 162 | 162 | 151 | 166 | 141 | 141 | 111 | 120 | 107 | 107 | 98 | 98 | 144 | 161 | 45 |
| SER044 | Soroti | Soroti | 2002-2003 | 162 | 176 | 151 | 166 | 141 | 141 | 111 | 120 | 107 | 107 | 98 | 98 | 144 | 144 | 46 |
| SER060 | Soroti | Soroti | 2002-2003 | 162 | 176 | 151 | 166 | 115 | 141 | 111 | 120 | 107 | 107 | 98 | 98 | 144 | 161 | 47 |
| SER002 | Soroti | Soroti | 2002-2003 | 162 | 176 | 151 | 166 | 141 | 141 | 111 | 120 | 107 | 107 | 98 | 98 | 144 | 161 | 49 |
| SER008 | Soroti | Soroti | 2002-2003 | 162 | 176 | 151 | 166 | 141 | 141 | 111 | 120 | 107 | 107 | 98 | 98 | 144 | 161 | 49 |
| SER009 | Soroti | Soroti | 2002-2003 | 162 | 176 | 151 | 166 | 141 | 141 | 111 | 120 | 107 | 107 | 98 | 98 | 144 | 161 | 49 |
| SER010 | Soroti | Soroti | 2002-2003 | 162 | 176 | 151 | 166 | 141 | 141 | 111 | 120 | 107 | 107 | 98 | 98 | 144 | 161 | 49 |
| SER011 | Soroti | Soroti | 2002-2003 | 162 | 176 | 151 | 166 | 141 | 141 | 111 | 120 | 107 | 107 | 98 | 98 | 144 | 161 | 49 |
| SER012 | Soroti | Soroti | 2002-2003 | 162 | 176 | 151 | 166 | 141 | 141 | 111 | 120 | 107 | 107 | 98 | 98 | 144 | 161 | 49 |
| SER013 | Soroti | Soroti | 2002-2003 | 162 | 176 | 151 | 166 | 141 | 141 | 111 | 120 | 107 | 107 | 98 | 98 | 144 | 161 | 49 |
| SER014 | Soroti | Soroti | 2002-2003 | 162 | 176 | 151 | 166 | 141 | 141 | 111 | 120 | 107 | 107 | 98 | 98 | 144 | 161 | 49 |
| SER017 | Soroti | Soroti | 2002-2003 | 162 | 176 | 151 | 166 | 141 | 141 | 111 | 120 | 107 | 107 | 98 | 98 | 144 | 161 | 49 |
| SER018 | Soroti | Soroti | 2002-2003 | 162 | 176 | 151 | 166 | 141 | 141 | 111 | 120 | 107 | 107 | 98 | 98 | 144 | 161 | 49 |
| SER022 | Soroti | Soroti | 2002-2003 | 162 | 176 | 151 | 166 | 141 | 141 | 111 | 120 | 107 | 107 | 98 | 98 | 144 | 161 | 49 |
| SER023 | Soroti | Soroti | 2002-2003 | 162 | 176 | 151 | 166 | 141 | 141 | 111 | 120 | 107 | 107 | 98 | 98 | 144 | 161 | 49 |
| SER025 | Soroti | Soroti | 2002-2003 | 162 | 176 | 151 | 166 | 141 | 141 | 111 | 120 | 107 | 107 | 98 | 98 | 144 | 161 | 49 |
| SER026 | Soroti | Soroti | 2002-2003 | 162 | 176 | 151 | 166 | 141 | 141 | 111 | 120 | 107 | 107 | 98 | 98 | 144 | 161 | 49 |
| SER028 | Soroti | Soroti | 2002-2003 | 162 | 176 | 151 | 166 | 141 | 141 | 111 | 120 | 107 | 107 | 98 | 98 | 144 | 161 | 49 |
| SER029 | Soroti | Soroti | 2002-2003 | 162 | 176 | 151 | 166 | 141 | 141 | 111 | 120 | 107 | 107 | 98 | 98 | 144 | 161 | 49 |
| SER030 | Soroti | Soroti | 2002-2003 | 162 | 176 | 151 | 166 | 141 | 141 | 111 | 120 | 107 | 107 | 98 | 98 | 144 | 161 | 49 |
| SER031 | Soroti | Soroti | 2002-2003 | 162 | 176 | 151 | 166 | 141 | 141 | 111 | 120 | 107 | 107 | 98 | 98 | 144 | 161 | 49 |
| SER032 | Soroti | Soroti | 2002-2003 | 162 | 176 | 151 | 166 | 141 | 141 | 111 | 120 | 107 | 107 | 98 | 98 | 144 | 161 | 49 |
| SER033 | Soroti | Soroti | 2002-2003 | 162 | 176 | 151 | 166 | 141 | 141 | 111 | 120 | 107 | 107 | 98 | 98 | 144 | 161 | 49 |
| SER036 | Soroti | Soroti | 2002-2003 | 162 | 176 | 151 | 166 | 141 | 141 | 111 | 120 | 107 | 107 | 98 | 98 | 144 | 161 | 49 |
| SER037 | Soroti | Soroti | 2002-2003 | 162 | 176 | 151 | 166 | 141 | 141 | 111 | 120 | 107 | 107 | 98 | 98 | 144 | 161 | 49 |
| SER039 | Soroti | Soroti | 2002-2003 | 162 | 176 | 151 | 166 | 141 | 141 | 111 | 120 | 107 | 107 | 98 | 98 | 144 | 161 | 49 |
| SER040 | Soroti | Soroti | 2002-2003 | 162 | 176 | 151 | 166 | 141 | 141 | 111 | 120 | 107 | 107 | 98 | 98 | 144 | 161 | 49 |
| SER045 | Soroti | Soroti | 2002-2003 | 162 | 176 | 151 | 166 | 141 | 141 | 111 | 120 | 107 | 107 | 98 | 98 | 144 | 161 | 49 |
| SER048 | Soroti | Soroti | 2002-2003 | 162 | 176 | 151 | 166 | 141 | 141 | 111 | 120 | 107 | 107 | 98 | 98 | 144 | 161 | 49 |
| SER049 | Soroti | Soroti | 2002-2003 | 162 | 176 | 151 | 166 | 141 | 141 | 111 | 120 | 107 | 107 | 98 | 98 | 144 | 161 | 49 |
| SER051 | Soroti | Soroti | 2002-2003 | 162 | 176 | 151 | 166 | 141 | 141 | 111 | 120 | 107 | 107 | 98 | 98 | 144 | 161 | 49 |
| SER053 | Soroti | Soroti | 2002-2003 | 162 | 176 | 151 | 166 | 141 | 141 | 111 | 120 | 107 | 107 | 98 | 98 | 144 | 161 | 49 |
| SER055 | Soroti | Soroti | 2002-2003 | 162 | 176 | 151 | 166 | 141 | 141 | 111 | 120 | 107 | 107 | 98 | 98 | 144 | 161 | 49 |
| SER056 | Soroti | Soroti | 2002-2003 | 162 | 176 | 151 | 166 | 141 | 141 | 111 | 120 | 107 | 107 | 98 | 98 | 144 | 161 | 49 |
| SER061 | Soroti | Soroti | 2002-2003 | 162 | 176 | 151 | 166 | 141 | 141 | 111 | 120 | 107 | 107 | 98 | 98 | 144 | 161 | 49 |
| SER063 | Soroti | Soroti | 2002-2003 | 162 | 176 | 151 | 166 | 141 | 141 | 111 | 120 | 107 | 107 | 98 | 98 | 144 | 161 | 49 |
| SER065 | Soroti | Soroti | 2002-2003 | 162 | 176 | 151 | 166 | 141 | 141 | 111 | 120 | 107 | 107 | 98 | 98 | 144 | 161 | 49 |
| SER068 | Soroti | Soroti | 2002-2003 | 162 | 176 | 151 | 166 | 141 | 141 | 111 | 120 | 107 | 107 | 98 | 98 | 144 | 161 | 49 |
| SER069 | Soroti | Soroti | 2002-2003 | 162 | 176 | 151 | 166 | 141 | 141 | 111 | 120 | 107 | 107 | 98 | 98 | 144 | 161 | 49 |
| SER071 | Soroti | Soroti | 2002-2003 | 162 | 176 | 151 | 166 | 141 | 141 | 111 | 120 | 107 | 107 | 98 | 98 | 144 | 161 | 49 |
| SER072 | Soroti | Soroti | 2002-2003 | 162 | 176 | 151 | 166 | 141 | 141 | 111 | 120 | 107 | 107 | 98 | 98 | 144 | 161 | 49 |
| SER073 | Soroti | Soroti | 2002-2003 | 162 | 176 | 151 | 166 | 141 | 141 | 111 | 120 | 107 | 107 | 98 | 98 | 144 | 161 | 49 |
| SER074 | Soroti | Soroti | 2002-2003 | 162 | 176 | 151 | 166 | 141 | 141 | 111 | 120 | 107 | 107 | 98 | 98 | 144 | 161 | 49 |
| SER075 | Soroti | Soroti | 2002-2003 | 162 | 176 | 151 | 166 | 141 | 141 | 111 | 120 | 107 | 107 | 98 | 98 | 144 | 161 | 49 |
| SER076 | Soroti | Soroti | 2002-2003 | 162 | 176 | 151 | 166 | 141 | 141 | 111 | 120 | 107 | 107 | 98 | 98 | 144 | 161 | 49 |
| SER078 | Soroti | Soroti | 2002-2003 | 162 | 176 | 151 | 166 | 141 | 141 | 111 | 120 | 107 | 107 | 98 | 98 | 144 | 161 | 49 |
| SER080 | Soroti | Soroti | 2002-2003 | 162 | 176 | 151 | 166 | 141 | 141 | 111 | 120 | 107 | 107 | 98 | 98 | 144 | 161 | 49 |
| SER082 | Soroti | Soroti | 2002-2003 | 162 | 176 | 151 | 166 | 141 | 141 | 111 | 120 | 107 | 107 | 98 | 98 | 144 | 161 | 49 |
| SER083 | Soroti | Soroti | 2002-2003 | 162 | 176 | 151 | 166 | 141 | 141 | 111 | 120 | 107 | 107 | 98 | 98 | 144 | 161 | 49 |
| SER084 | Soroti | Soroti | 2002-2003 | 162 | 176 | 151 | 166 | 141 | 141 | 111 | 120 | 107 | 107 | 98 | 98 | 144 | 161 | 49 |
| SER085 | Soroti | Soroti | 2002-2003 | 162 | 176 | 151 | 166 | 141 | 141 | 111 | 120 | 107 | 107 | 98 | 98 | 144 | 161 | 49 |
| SER086 | Soroti | Soroti | 2002-2003 | 162 | 176 | 151 | 166 | 141 | 141 | 111 | 120 | 107 | 107 | 98 | 98 | 144 | 161 | 49 |
| SER087 | Soroti | Soroti | 2002-2003 | 162 | 176 | 151 | 166 | 141 | 141 | 111 | 120 | 107 | 107 | 98 | 98 | 144 | 161 | 49 |
| SER004 | Soroti | Soroti | 2002-2003 | 162 | 176 | 151 | 166 | 141 | 141 | 111 | 120 | 107 | 107 | 98 | 98 | 144 | 164 | 50 |
| LIRI003 | Soroti | Soroti | 2002-2003 | 162 | 162 | 151 | 166 | 141 | 141 | 111 | 120 | 107 | 107 | 98 | 108 | 144 | 155 | 51 |
| SER108 | Soroti | Soroti | 2002-2003 | 162 | 176 | 151 | 166 | 141 | 141 | 111 | 120 | 107 | 107 | 98 | 124 | 144 | 161 | 52 |
| SER059 | Soroti | Soroti | 2002-2003 | 162 | 176 | 151 | 166 | 141 | 141 | 111 | 120 | 107 | 134 | 98 | 98 | 144 | 161 | 56 |
| SER054 | Soroti | Soroti | 2002-2003 | 0 | 0 | 151 | 166 | 141 | 141 | 111 | 120 | 107 | 107 | 98 | 98 | 144 | 161 |  |
| SER070 | Soroti | Soroti | 2002-2003 | 0 | 0 | 151 | 166 | 141 | 141 | 111 | 120 | 107 | 107 | 98 | 98 | 144 | 161 |  |
| SER050 | Soroti | Soroti | 2002-2003 | 162 | 176 | 0 | 0 | 141 | 141 | 111 | 120 | 107 | 107 | 98 | 98 | 144 | 161 |  |
| SER081 | Soroti | Soroti | 2002-2003 | 162 | 176 | 151 | 166 | 141 | 141 | 111 | 120 | 107 | 107 | 0 | 0 | 144 | 161 |  |
| LIRI001 | Tororo | Tororo | 2002-2003 | 162 | 162 | 124 | 151 | 122 | 141 | 111 | 120 | 98 | 107 | 98 | 98 | 144 | 161 | 13 |
| LIRI033 | Tororo | Tororo | 2002-2003 | 162 | 176 | 151 | 151 | 115 | 138 | 111 | 120 | 98 | 107 | 90 | 98 | 144 | 150 | 22 |
| LIRI029 | Tororo | Tororo | 2002-2003 | 162 | 176 | 151 | 151 | 122 | 122 | 111 | 120 | 98 | 107 | 90 | 98 | 144 | 161 | 23 |
| LIRI005 | Tororo | Tororo | 2002-2003 | 162 | 176 | 151 | 151 | 122 | 141 | 111 | 120 | 98 | 107 | 90 | 98 | 144 | 161 | 24 |
| LIRI018 | Tororo | Tororo | 2002-2003 | 162 | 176 | 151 | 151 | 122 | 141 | 111 | 120 | 98 | 107 | 90 | 98 | 144 | 161 | 24 |
| LIRI030 | Tororo | Tororo | 2002-2003 | 162 | 176 | 151 | 151 | 122 | 141 | 111 | 120 | 98 | 107 | 90 | 98 | 144 | 161 | 24 |
| LIRI031 | Tororo | Tororo | 2002-2003 | 162 | 176 | 151 | 151 | 122 | 141 | 111 | 120 | 98 | 107 | 90 | 98 | 144 | 161 | 24 |
| LIRI032 | Tororo | Tororo | 2002-2003 | 162 | 176 | 151 | 151 | 122 | 141 | 111 | 120 | 98 | 107 | 90 | 98 | 144 | 161 | 24 |
| LIRI008 | Tororo | Tororo | 2002-2003 | 162 | 162 | 151 | 151 | 122 | 141 | 111 | 120 | 98 | 107 | 98 | 98 | 144 | 161 | 25 |
| LIRI022 | Tororo | Tororo | 2002-2003 | 162 | 162 | 151 | 151 | 122 | 141 | 111 | 120 | 98 | 107 | 98 | 98 | 144 | 161 | 25 |
| LIRI016 | Tororo | Tororo | 2002-2003 | 162 | 176 | 151 | 151 | 141 | 141 | 111 | 120 | 98 | 107 | 98 | 98 | 144 | 155 | 26 |
| LIRI002 | Tororo | Tororo | 2002-2003 | 162 | 176 | 151 | 151 | 122 | 141 | 111 | 120 | 98 | 107 | 98 | 98 | 144 | 161 | 27 |
| LIRI004 | Tororo | Tororo | 2002-2003 | 162 | 176 | 151 | 151 | 122 | 141 | 111 | 120 | 98 | 107 | 98 | 98 | 144 | 161 | 27 |
| LIRI012 | Tororo | Tororo | 2002-2003 | 162 | 176 | 151 | 151 | 122 | 141 | 111 | 120 | 98 | 107 | 98 | 98 | 144 | 161 | 27 |
| LIRI028 | Tororo | Tororo | 2002-2003 | 162 | 176 | 151 | 151 | 122 | 141 | 111 | 120 | 98 | 107 | 98 | 98 | 144 | 161 | 27 |
| LIRI025 | Tororo | Tororo | 2002-2003 | 162 | 176 | 151 | 166 | 141 | 141 | 111 | 120 | 98 | 107 | 90 | 98 | 144 | 161 | 29* |
| LIRI015 | Tororo | Tororo | 2002-2003 | 162 | 176 | 151 | 166 | 141 | 141 | 111 | 120 | 98 | 107 | 98 | 98 | 144 | 161 | 31* |
| LIRI011 | Tororo | Tororo | 2002-2003 | 162 | 162 | 151 | 151 | 122 | 141 | 111 | 120 | 107 | 107 | 90 | 98 | 144 | 161 | 39 |
| LIRI024 | Tororo | Tororo | 2002-2003 | 162 | 176 | 151 | 151 | 122 | 122 | 111 | 120 | 107 | 107 | 98 | 98 | 144 | 161 | 41 |
| LIRI010 | Tororo | Tororo | 2002-2003 | 162 | 162 | 151 | 151 | 122 | 141 | 111 | 120 | 107 | 152 | 90 | 98 | 144 | 161 | 57 |
| LIRI023 | Tororo | Tororo | 2002-2003 | 162 | 162 | 151 | 151 | 122 | 141 | 111 | 120 | 107 | 152 | 90 | 98 | 144 | 161 | 57 |
| LIRI007 | Tororo | Tororo | 2002-2003 | 162 | 176 | 151 | 151 | 122 | 141 | 120 | 120 | 98 | 107 | 90 | 98 | 144 | 161 | 58 |
| LIRI014 | Tororo | Tororo | 2002-2003 | 162 | 162 | 151 | 151 | 122 | 141 | 120 | 120 | 98 | 107 | 98 | 98 | 144 | 155 | 59 |
| LIRI019 | Tororo | Tororo | 2002-2003 | 162 | 162 | 151 | 151 | 141 | 141 | 120 | 120 | 98 | 107 | 98 | 108 | 144 | 155 | 60 |
| LIRI009 | Tororo | Tororo | 2002-2003 | 162 | 176 | 151 | 166 | 122 | 141 | 120 | 120 | 98 | 107 | 90 | 98 | 144 | 161 | 61 |
| LIRI013 | Tororo | Tororo | 2002-2003 | 162 | 176 | 151 | 166 | 122 | 141 | 120 | 120 | 98 | 107 | 98 | 98 | 144 | 161 | 62 |
| LIRI027 | Tororo | Tororo | 2002-2003 | 162 | 162 | 151 | 151 | 0 | 0 | 111 | 120 | 98 | 107 | 90 | 98 | 0 | 0 |  |
| LIRI026 | Tororo | Tororo | 2002-2003 | 162 | 176 | 151 | 151 | 0 | 0 | 111 | 120 | 98 | 107 | 90 | 98 | 144 | 161 |  |
| LIRI021 | Tororo | Tororo | 2002-2003 | 162 | 176 | 151 | 151 | 0 | 0 | 111 | 120 | 107 | 122 | 90 | 98 | 144 | 164 |  |
| K3192 | Ug/Ke61-97 | Busia | 1989 | 162 | 162 | 143 | 157 | 138 | 141 | 111 | 120 | 107 | 107 | 108 | 108 | 144 | 155 | 37 |
| Mela80 | Ug/Ke 61-97 | Tororo | 1990 | 162 | 219 | 148 | 151 | 122 | 141 | 111 | 120 | 107 | 107 | 98 | 98 | 150 | 155 | 38 |
| K3442 | Ug/Ke 61-97 | Busia | 1997 | 162 | 176 | 151 | 151 | 120 | 141 | 111 | 120 | 107 | 107 | 98 | 98 | 144 | 161 | 40 |
| K3438 | Ug/Ke 61-97 | Busia | 1997 | 162 | 176 | 151 | 166 | 122 | 141 | 111 | 120 | 107 | 107 | 98 | 98 | 144 | 161 | 48 |
| K3198 | Ug/Ke 61-97 | Busia | 1988 | 162 | 162 | 157 | 157 | 138 | 141 | 111 | 120 | 107 | 107 | 98 | 98 | 144 | 155 | 53 |
| K3440 | Ug/Ke 61-97 | Busia | 1997 | 162 | 176 | 157 | 157 | 120 | 141 | 111 | 120 | 107 | 107 | 98 | 98 | 144 | 161 | 54 |
| K3448 | Ug/Ke 61-97 | Busia | Unknown^$^ | 162 | 176 | 157 | 157 | 120 | 141 | 111 | 120 | 107 | 107 | 98 | 98 | 144 | 161 | 54 |
| K2340 | Ug/Ke 61-97 | Busia | 1977 | 162 | 162 | 157 | 157 | 138 | 141 | 111 | 120 | 107 | 107 | 108 | 108 | 144 | 155 | 55 |
| EA2498 | Ug/Ke 61-97 | Tororo | 1977 | 162 | 162 | 143 | 151 | 138 | 141 | 120 | 120 | 107 | 107 | 98 | 108 | 144 | 155 | 63 |
| K3183 | Ug/Ke 61-97 | Busia | 1988 | 162 | 162 | 148 | 157 | 138 | 141 | 120 | 120 | 107 | 107 | 98 | 98 | 144 | 155 | 64 |
| K2976 | Ug/Ke 61-97 | Busia | 1987 | 162 | 162 | 157 | 157 | 138 | 141 | 120 | 120 | 107 | 107 | 98 | 98 | 144 | 155 | 65 |
| K3176 | Ug/Ke 61-97 | Busia | 1988 | 162 | 162 | 157 | 157 | 138 | 141 | 120 | 120 | 107 | 107 | 98 | 98 | 144 | 155 | 65 |
| K3180 | Ug/Ke 61-97 | Busia | 1988 | 162 | 162 | 157 | 157 | 138 | 141 | 120 | 120 | 107 | 107 | 98 | 98 | 144 | 155 | 65 |
| K3196 | Ug/Ke 61-97 | Busia | 1989 | 162 | 162 | 157 | 157 | 138 | 141 | 120 | 120 | 107 | 107 | 98 | 98 | 144 | 155 | 65 |
| UgA90 | Ug/Ke 61-97 | Busoga | 1990 | 162 | 162 | 157 | 157 | 138 | 141 | 120 | 120 | 107 | 107 | 98 | 98 | 144 | 155 | 65 |
| UgL | Ug/Ke 61-97 | Busoga | 1988 | 162 | 162 | 157 | 157 | 138 | 141 | 120 | 120 | 107 | 107 | 98 | 98 | 144 | 155 | 65 |
| K3199 | Ug/Ke 61-97 | Busia | 1989 | 162 | 162 | 157 | 157 | 120 | 154 | 120 | 120 | 107 | 107 | 98 | 108 | 144 | 155 | 66 |
| K3203 | Ug/Ke 61-97 | Busia | 1989 | 162 | 162 | 157 | 157 | 138 | 141 | 120 | 120 | 107 | 107 | 98 | 108 | 144 | 155 | 67 |
| K3205 | Ug/Ke 61-97 | Busia | 1989 | 162 | 162 | 157 | 157 | 138 | 141 | 120 | 120 | 107 | 107 | 98 | 108 | 144 | 155 | 67 |
| K3206 | Ug/Ke 61-97 | Busia | 1989 | 162 | 162 | 157 | 157 | 138 | 141 | 120 | 120 | 107 | 107 | 98 | 108 | 144 | 155 | 67 |
| UgM | Ug/Ke 61-97 | Busoga | 1988 | 162 | 162 | 157 | 157 | 138 | 141 | 120 | 120 | 107 | 107 | 98 | 108 | 144 | 155 | 67 |
| K3188 | Ug/Ke 61-97 | Busia | Unknown^$^ | 162 | 162 | 157 | 157 | 138 | 141 | 120 | 120 | 107 | 107 | 108 | 108 | 144 | 144 | 68 |
| K3189 | Ug/Ke 61-97 | Busia | Unknown^$^ | 162 | 162 | 157 | 157 | 138 | 141 | 120 | 120 | 107 | 107 | 108 | 108 | 144 | 144 | 68 |
| K2344 | Ug/Ke 61-97 | Busia | 1977 | 162 | 162 | 157 | 157 | 138 | 141 | 120 | 120 | 107 | 107 | 108 | 108 | 144 | 155 | 69 |
| K2427 | Ug/Ke 61-97 | Busia | 1979 | 162 | 162 | 157 | 157 | 138 | 141 | 120 | 120 | 107 | 107 | 108 | 108 | 144 | 155 | 69 |
| K2428 | Ug/Ke 61-97 | Busia | 1979 | 162 | 162 | 157 | 157 | 138 | 141 | 120 | 120 | 107 | 107 | 108 | 108 | 144 | 155 | 69 |
| K2556 | Ug/Ke 61-97 | Busia | 1982 | 162 | 162 | 157 | 157 | 138 | 141 | 120 | 120 | 107 | 107 | 108 | 108 | 144 | 155 | 69 |
| K3185 | Ug/Ke 61-97 | Busia | 1997 | 162 | 162 | 157 | 157 | 138 | 141 | 120 | 120 | 107 | 107 | 108 | 108 | 144 | 155 | 69 |
| K3190 | Ug/Ke 61-97 | Busia | 1989 | 162 | 162 | 157 | 157 | 138 | 141 | 120 | 120 | 107 | 107 | 108 | 108 | 144 | 155 | 69 |
| K3186 | Ug/Ke 61-97 | Busia | 1989 | 162 | 162 | 157 | 157 | 141 | 138 | 120 | 120 | 107 | 107 | 108 | 108 | 144 | 155 | 70 |
| K2381 | Ug/Ke 61-97 | Busia | 1978 | 162 | 162 | 157 | 157 | 141 | 141 | 120 | 120 | 107 | 107 | 108 | 108 | 144 | 155 | 71 |
| K2382 | Ug/Ke 61-97 | Busia | 1978 | 162 | 162 | 157 | 157 | 141 | 141 | 120 | 120 | 107 | 107 | 108 | 108 | 144 | 155 | 71 |
| UgC90 | Ug/Ke 61-97 | Busoga | 1990 | 162 | 162 | 157 | 166 | 115 | 138 | 120 | 120 | 107 | 107 | 98 | 10 | 155 | 161 | 72 |
| 3200 | Ug/Ke 61-97 | Busia | 1989 | 162 | 162 | 157 | 166 | 138 | 141 | 120 | 120 | 107 | 107 | 98 | 98 | 144 | 155 | 73 |
| U89/8 | Ug/Ke 61-97 | Busoga | 1989 | 162 | 162 | 157 | 166 | 138 | 141 | 120 | 120 | 107 | 107 | 98 | 98 | 144 | 155 | 73 |
| UgI | Ug/Ke 61-97 | Busoga | 1988 | 162 | 162 | 157 | 166 | 138 | 141 | 120 | 120 | 107 | 107 | 98 | 98 | 144 | 155 | 73 |
| UgJ | Ug/Ke 61-97 | Busoga | 1988 | 162 | 162 | 157 | 166 | 138 | 141 | 120 | 120 | 107 | 107 | 98 | 98 | 144 | 155 | 73 |
| UTAR3 | Ug/Ke 61-97 | Busoga | 1981 | 162 | 162 | 157 | 166 | 136 | 141 | 120 | 120 | 107 | 107 | 98 | 108 | 144 | 155 | 74 |
| 1042 | Ug/Ke 61-97 | Nyanza | 1961 | 162 | 162 | 157 | 166 | 138 | 141 | 120 | 120 | 107 | 107 | 98 | 108 | 144 | 155 | 75 |
| 1301 | Ug/Ke 61-97 | Nyanza | 1961 | 162 | 162 | 157 | 166 | 138 | 141 | 120 | 120 | 107 | 107 | 98 | 108 | 144 | 155 | 75 |
| 3194 | Ug/Ke 61-97 | Busia | 1989 | 162 | 162 | 157 | 166 | 138 | 141 | 120 | 120 | 107 | 107 | 98 | 108 | 144 | 155 | 75 |
| 3206 | Ug/Ke 61-97 | Busia | 1989 | 162 | 162 | 157 | 166 | 138 | 141 | 120 | 120 | 107 | 107 | 98 | 108 | 144 | 155 | 75 |
| EATRO 795 | Ug/Ke 61-97 | Nyanza | 1964 | 162 | 162 | 157 | 166 | 138 | 141 | 120 | 120 | 107 | 107 | 98 | 108 | 144 | 155 | 75 |
| K3445 | Ug/Ke 61-97 | Busia | 1997 | 224 | 237 | 148 | 157 | 136 | 138 | 111 | 111 | 107 | 107 | 0 | 0 | 150 | 161 |  |
| K2380 | Ug/Ke 61-97 | Busia | 1978 | 162 | 162 | 0 | 0 | 141 | 141 | 120 | 120 | 0 | 0 | 108 | 108 | 0 | 0 |  |
| MA66 | Ug/Ke 61-97 | Iyowla | 1988 | 162 | 162 | 0 | 0 | 138 | 141 | 120 | 120 | 107 | 107 | 0 | 0 | 144 | 155 |  |
| K3110 | Ug/Ke 61-97 | Busia | 1989 | 162 | 162 | 157 | 157 | 122 | 141 | 120 | 120 | 107 | 107 | 86 | 86 | 0 | 0 |  |
| K2350 | Ug/Ke 61-97 | Busia | 1977 | 162 | 162 | 157 | 157 | 138 | 141 | 120 | 120 | 107 | 107 | 108 | 108 | 0 | 0 |  |
| MAWERA66 | Ug/Ke 61-97 | Mawero | 1990 | 162 | 162 | 151 | 151 | 138 | 141 | 120 | 120 | 107 | 107 | 0 | 0 | 144 | 155 |  |
| K116 | Ug/Ke 61-97 | Iyowla | 1990 | 162 | 162 | 157 | 157 | 138 | 141 | 120 | 120 | 107 | 107 | 0 | 0 | 144 | 155 |  |

^$^Exact date of sampling has not been recorded for these samples; due to the collections they came from we are confident the sampling date falls within the period 1961-1997.
