# Supplementary material for: Population Genetics of Trypanosoma brucei rhodesiense: Clonality and Diversity within and between Foci
Source: PLoS Negl Trop Dis. 2013 Nov 14;7(11):e2526. doi: 10.1371/journal.pntd.0002526 (PMC3828156; doi:10.1371/journal.pntd.0002526)
Supplement: Table S2 — Microsatellite loci and the primers used for their amplification. For each locus the first pair of primers were used for the primary reaction and the second pair for the subsequent nested reaction. (DOCX) [file pntd.0002526.s002.docx]

| Microsatellite ID | Chromosome | Primer ID | 5’ – 3’ Sequence |
| --- | --- | --- | --- |
| Ch1/18 | 1 | CH1/18-C  Ch1/18-D  *CH1/18-A*  *CH1/18-B* | TATAATGCGTTTGTGAGAAT  GAAGGGAGGGAACAGAAGCAGGG  *TGTGAGAATGGTACTCACGCGCTG*  *ACAACGTTAGCACACAATTCCTGTG* |
| Ch2/PLC | 2 | CH2/PLC-G2  CH2/PLC-H4  *CH2/PLC-G*  *CH2/PLC-H3* | TTAAGTGGACGACGAAATAACAACA  TTCAAACACCGTCCCCCTCAATAAT  *CAACGACGTTGGAAGAGTGTGAAC*  *CCACTGACCTTTCATTTGATCGCTTTC* |
| Ch3/5L5 | 3 | CH3/5L5-AA  CH3/5L5-B  *CH3/5L5-A*  *CH3/5L5-BB* | GAGCGTACATTGCAGGTAGTGCGTAGCG  GGAAACTGCTTAAACTTGCGTGAG  *GTACGTGGTTAACCACAACCTACT*  *GTATTTTTCATGGCACACAACATAT* |
| Ch3/IJ15/1 | 3 | Ch3/IJ15/1-C  Ch3/IJ15/1-D  *Ch3/IJ15/1-A*  *Ch3/IJ15/1-B* | AGGCTTAGACGAGTGTCAGG  GTAAATAGACACAGTGAAACCG  *GTTAGGTTACGCAAGTCAGT*  *GAAACACTCAGTTCCACACC* |
| Ch4/M12C12 | 4 | CH4/M12C12-B  CH4/M12C12-C  *CH4/M12C12-A*  *CH4/M12C12-D* | TACCCTCATCAAGTGGTCG  AAAACCTCATCCAGTCGCACTGG  *TGGACACACAGAAGCCTACCG*  *AGTGTGGTGGTGCGTGCAAACTTGG* |
| Ch5/JS2 | 5 | CH5/JS2-C  CH5/JS2-D  *CH5/JS2-A*  *CH5/JS2-B* | AGTAATGGGAATGAGCGTCACCAG  GATCTTCGCTTACACAAGCGGTAC  *GATTGGCGCAACAACTTTCACATACG*  *CCCTTTCTTCCTTGGCCATTGTTTTACTAT* |
| Ch9/4 | 9 | Ch9/4-C  Ch9/4-D  *Ch9/4-A*  *Ch9/4-B* | CATCGATGAGAAGTACACTG  AACAGACTAGGAAAGTATAC  *GTGGAGGAGTGCTGATGA*  *ATGTAAGATATTAGAGCAGTAAA* |

Table S2. Microsatellite loci and the primers used for their amplification. For each locus the first pair of primers were used for the primary reaction and the second pair for the subsequent nested reaction.
