# Supplementary material for: Population Genetics of Trypanosoma brucei rhodesiense: Clonality and Diversity within and between Foci
Source: PLoS Negl Trop Dis. 2013 Nov 14;7(11):e2526. doi: 10.1371/journal.pntd.0002526 (PMC3828156; doi:10.1371/journal.pntd.0002526)
Supplement: Table S3 — Allele frequencies for the seven microsatellite markers in all four trypanosome populations. (DOCX) [file pntd.0002526.s003.docx]

Table S3. Allele frequencies for the seven microsatellite markers in all four trypanosome populations.

| **Locus** | **Allele/n** | **Ug/Ke 61-97** | **Tororo** | **Soroti** | **Malawi** |
| --- | --- | --- | --- | --- | --- |
| **Ch1/18** | **N** | 50 | 29 | 82 | 28 |
|  | **1** | 0.930 | 0.655 | 0.512 | 0.893 |
|  | **3** | 0.010 | 0.000 | 0.000 | 0.000 |
|  | **4** | 0.040 | 0.345 | 0.488 | 0.000 |
|  | **5** | 0.000 | 0.000 | 0.000 | 0.018 |
|  | **6** | 0.000 | 0.000 | 0.000 | 0.089 |
|  | **7** | 0.010 | 0.000 | 0.000 | 0.000 |
|  | **9** | 0.010 | 0.000 | 0.000 | 0.000 |
| **Ch2/PLC** | **N** | 48 | 29 | 83 | 28 |
|  | **1** | 0.021 | 0.000 | 0.006 | 0.054 |
|  | **2** | 0.031 | 0.000 | 0.000 | 0.036 |
|  | **4** | 0.000 | 0.000 | 0.018 | 0.893 |
|  | **5** | 0.000 | 0.017 | 0.000 | 0.018 |
|  | **6** | 0.073 | 0.914 | 0.548 | 0.000 |
|  | **7** | 0.125 | 0.069 | 0.428 | 0.000 |
|  | **8** | 0.750 | 0.000 | 0.000 | 0.000 |
| **Ch3/5L5** | **N** | 50 | 29 | 84 | 28 |
|  | **1** | 0.100 | 0.414 | 0.500 | 0.768 |
|  | **2** | 0.900 | 0.586 | 0.500 | 0.232 |
| **Ch3/IJ15/1** | **N** | 50 | 26 | 84 | 26 |
|  | **1** | 0.020 | 0.000 | 0.000 | 0.000 |
|  | **2** | 0.010 | 0.019 | 0.006 | 0.173 |
|  | **3** | 0.000 | 0.000 | 0.000 | 0.385 |
|  | **5** | 0.010 | 0.000 | 0.000 | 0.000 |
|  | **7** | 0.000 | 0.000 | 0.000 | 0.365 |
|  | **8** | 0.040 | 0.000 | 0.000 | 0.077 |
|  | **9** | 0.500 | 0.519 | 0.994 | 0.000 |
|  | **10** | 0.030 | 0.442 | 0.000 | 0.000 |
|  | **13** | 0.390 | 0.019 | 0.000 | 0.000 |
| **Ch4/M12** | **N** | 49 | 29 | 84 | 28 |
|  | **1** | 0.000 | 0.414 | 0.071 | 0.357 |
|  | **2** | 1.000 | 0.534 | 0.923 | 0.643 |
|  | **3** | 0.000 | 0.034 | 0.000 | 0.000 |
|  | **4** | 0.000 | 0.017 | 0.000 | 0.000 |
|  | **5** | 0.000 | 0.000 | 0.006 | 0.000 |
| **Ch5/JS2** | **N** | 46 | 29 | 83 | 28 |
|  | **2** | 0.000 | 0.000 | 0.000 | 0.357 |
|  | **4** | 0.511 | 0.707 | 0.970 | 0.625 |
|  | **5** | 0.457 | 0.017 | 0.006 | 0.000 |
|  | **6** | 0.000 | 0.000 | 0.006 | 0.000 |
|  | **8** | 0.000 | 0.276 | 0.018 | 0.018 |
|  | **10** | 0.011 | 0.000 | 0.000 | 0.000 |
|  | **23** | 0.022 | 0.000 | 0.000 | 0.000 |
| **Ch9/4** | **N** | 47 | 28 | 84 | 24 |
|  | **1** | 0.064 | 0.411 | 0.476 | 0.000 |
|  | **2** | 0.000 | 0.000 | 0.000 | 0.583 |
|  | **5** | 0.489 | 0.500 | 0.506 | 0.042 |
|  | **6** | 0.000 | 0.018 | 0.006 | 0.000 |
|  | **7** | 0.426 | 0.054 | 0.006 | 0.000 |
|  | **9** | 0.000 | 0.000 | 0.000 | 0.375 |
|  | **10** | 0.021 | 0.018 | 0.000 | 0.000 |
|  | **11** | 0.000 | 0.000 | 0.006 | 0.000 |
