# Supplementary material for: Population Genetics of Trypanosoma brucei rhodesiense: Clonality and Diversity within and between Foci
Source: PLoS Negl Trop Dis. 2013 Nov 14;7(11):e2526. doi: 10.1371/journal.pntd.0002526 (PMC3828156; doi:10.1371/journal.pntd.0002526)
Supplement: Table S4 — Linkage disequilibrium between pairs of loci for the two populations where analysis was warranted for ‘all samples/unique MLGs’, respectively. Allele combinations were preserved for loci showing significant disagreement with HWE predictions. *P<0.05 = Significant linkage disequilibrium, indicated in bold. (DOCX) [file pntd.0002526.s004.docx]

Table S4. Linkage disequilibrium between pairs of loci for the two populations where analysis was warranted for ‘all samples/unique MLGs’, respectively. Allele combinations were preserved for loci showing significant disagreement with HWE predictions. *P < 0.05 = Significant linkage disequilibrium, indicated in bold.

**Ug/Ke 61-97**

|  | Ch3/5L5 | Ch4/M12C12 | Ch2/PLC | Ch5/JS2 | Ch1/18 | Ch9/4 | Ch3/IJ15/1 |
| --- | --- | --- | --- | --- | --- | --- | --- |
| Ch3/5L5 | - | 0.41 / 1.00 | **0.00 / 0.10** | 0.26 / 0.33 | **0.00 / 0.00** | **0.00 / 0.01** | **0.00 /** 0.06 |
| Ch4/M12C12 | - | - | 1.00 / 1.00 | 1.00 / 1.00 | 1.00 / 0.07 | 1.00 / 1.00 | 1.00 / 1.00 |
| Ch2/PLC | - | - | - | **0.03 /** 0.62 | **0.01 / 0.02** | **0.03 /** 0.19 | 0.09 / 0.42 |
| Ch5/JS2 | - | - | - | - | 0.19 / 0.45 | **0.00 /** 0.05 | **0.00 / 0.03** |
| Ch1/18 | - | - | - | - | - | **0.00 / 0.00** | **0.00 / 0.00** |
| Ch9/4 | - | - | - | - | - | - | **0.00** |
| Ch3/IJ15/1 | - | - | - | - | - | - | - |

**Malawi**

|  | Ch3/5L5 | Ch4/M12C12 | Ch2/PLC | Ch5/JS2 | Ch1/18 | Ch9/4 | Ch3/IJ15/1 |
| --- | --- | --- | --- | --- | --- | --- | --- |
| Ch3/5L5 | - | 0.11 / 0.81 | 0.61 / 0.43 | 0.64 / 0.52 | 0.55 / 0.69 | 0.87 / 0.57 | 0.25 / 0.42 |
| Ch4/M12C12 | - | - | 0.17 / 0.59 | 0.15 / 0.87 | 0.06 / 0.43 | 0.10 / 0.17 | 0.50 / 0.57 |
| Ch2/PLC | - | - | - | 0.79 / 0.89 | 0.70 / 0.70 | 0.29 / 0.50 | 0.18 / 0.24 |
| Ch5/JS2 | - | - | - | - | 0.46 / 0.75 | 0.28 / 0.57 | **0.02 /** 0.06 |
| Ch1/18 | - | - | - | - | - | 0.39 / 0.61 | 0.48 / 0.71 |
| Ch9/4 | - | - | - | - | - | - | **0.01 / 0.01** |
| Ch3/IJ15/1 | - | - | - | - | - | - | - |
